# Wnt-controlled sphingolipids modulate Anthrax Toxin Receptor palmitoylation to regulate oriented mitosis in zebrafish

**DOI:** 10.1038/s41467-020-17196-3

**Published:** 2020-07-03

**Authors:** I. Castanon, J. T. Hannich, L. Abrami, F. Huber, M. Dubois, M. Müller, F. G. van der Goot, M. Gonzalez-Gaitan

**Affiliations:** 1 Department of Biochemistry and NCCR Chemical Biology, 30 Quai Ernest-Ansermet, 1211 Geneva 4, Switzerland; 2 Global Health Institute, EPFL, Station 15, 1015 Lausanne, Switzerland; 3 Vital-IT, SIB Swiss Institute of Bioinformatics, Lausanne, Switzerland

**Keywords:** Biochemistry, Cell biology, Developmental biology

## Abstract

Oriented cell division is a fundamental mechanism to control asymmetric stem cell division, neural tube elongation and body axis extension, among other processes. During zebrafish gastrulation, when the body axis extends, dorsal epiblast cells display divisions that are robustly oriented along the animal-vegetal embryonic axis. Here, we use a combination of lipidomics, metabolic tracer analysis and quantitative image analysis to show that sphingolipids mediate spindle positioning during oriented division of epiblast cells. We identify the Wnt signaling as a regulator of sphingolipid synthesis that mediates the activity of serine palmitoyltransferase (SPT), the first and rate-limiting enzyme in sphingolipid production. Sphingolipids determine the palmitoylation state of the Anthrax receptor, which then positions the mitotic spindle of dividing epiblast cells. Our data show how Wnt signaling mediates sphingolipid-dependent oriented division and how sphingolipids determine Anthrax receptor palmitoylation, which ultimately controls the activation of Diaphanous to mediate spindle rotation and oriented mitosis.

## Abstract (web summary)

During development, oriented cell division is important to proper body axis extension. Here, the authors show that sphingolipids are required to direct spindle rotation and oriented mitosis via Anthrax receptor palmitoylation in zebrafish gastrulation.

## Introduction

The orientation of cell divisions is determined by the position of the mitotic spindle and plays crucial roles in early embryo body plan specification, axis determination, cell fate diversity, and in the morphogenesis of tissues and organs^1,2^. In particular, during zebrafish gastrulation, the divisions of epiblast cells are robustly oriented along the animal-vegetal (A/V) embryonic axis^3–5^ and can contribute to the extension of the axis^3,5^. We have previously shown that these cells form a polarized F-actin cap in metaphase that is aligned with respect to the embryonic axis, a process mediated by the Wnt/Planar Cell Polarity (PCP) pathway^5^. This cortical F-actin cap triggers the spatial redistribution of the transmembrane protein Anthrax toxin receptor 2a (Antxr2a), which becomes enriched at the F-actin cap and is required to position the mitotic spindle along the embryonic axis^5^. The orientation of the mitotic spindle by Antxr2a relies on its interaction with the small GTPase RhoA in its GTP-bound active state. This interaction is essential for the activity of the actin nucleator Diaphanous (Dia), which we found to be responsible to exert torque on the mitotic spindle to align it with the F-actin and Antxr2a caps and therefore with the embryonic axis^5^.

Anthrax toxin receptors as well as Wnt proteins and RhoA undergo post-translational lipid modifications, which have important implications in their functions in many cellular processes^6–8^. However, whether lipidation is important for their role in oriented division is still unclear. Protein lipidation typically results in membrane association and specific localization of lipidated proteins^9^. Indeed, the specific membrane localization and function of lipid-modified proteins depends in many instances on the membrane lipid composition^10^. There is growing evidence indicating that some membrane lipids, including sphingolipids, are essential for the establishment of polarity^11–13^. For instance, sphingolipids can cluster to provide a molecular platform by forming ordered membrane microdomains that are critical for protein sorting^14^. This has been shown to be important to promote cell polarity in both typical polarized epithelial cells^15^, as well as in transiently polarized cells such as migrating lymphocytes, neurons or mating yeast cells^16–18^. Based on this, we ought to study membrane lipids during Wnt/PCP-mediated oriented cell division.

In this study, we show that sphingolipids control the position of the mitotic spindle during oriented divisions of epiblast cells in zebrafish embryos. Lipidomics and metabolic tracer analysis reveal that the Wnt signaling pathway determines the sphingolipid composition of the plasma membrane by regulating the Serine Palmitoyltransferase (SPT) enzyme, which catalyzes the first and rate-limiting step of the de novo sphingolipid pathway^19^. We find that sphingolipids mediate mitotic spindle positioning by regulating the palmitoylation state of the Anthrax toxin receptor, which in turn is critical for the activation of Diaphanous to mediate the rotation of the spindle during oriented mitosis of epiblast cells.

## Results

### Lipid profile of zebrafish embryos at gastrula stages

We first examined the lipid profile of wild-type zebrafish embryos at gastrula stages (Supplementary Fig. 1) by applying in zebrafish a direct infusion tandem mass spectrometry (MS/MS) approach using multiple reaction monitoring^20^ (see “Methods”). This approach allowed us to identify and robustly quantify a great number of membrane lipids. This precise quantitative analysis uncovered a role for the Wnt/PCP pathway in the control of the embryonic levels of sphingolipids.

At early stages, the development of zebrafish embryos is partially sustained by a large, lipid-rich, yolk cell where the mother deposited lipids and lipid metabolism enzymes, which contribute to feed the embryo^21,22^. This maternally supplied pool of nutrients is, however, insufficient to power all the earliest developmental events^23^, implying an essential role for zygotically expressed lipid enzymes during early embryogenesis. Therefore, we first determined the spectrum of membrane lipid species in the yolk cell versus the deyolked embryo, independently (Supplementary Fig. 1a, b). Our approach allowed us to examine the abundance of around 700 membrane lipid species, grouped into two main lipid classes: sphingolipids (SL) and phospholipids, the latter including phosphatidylcholine (PC), phosphatidylinositol (PI), phosphatidylserine (PS), and phosphatidylethanolamine (PE).

A two-dimensional, interactive treemap representation of the lipid composition of zebrafish embryos showed that the lipidome of the deyolked embryo and in the yolk cell is rather similar (Supplementary Fig. 1c; [https://zebrafish-embryo-lipidome.vital-it.ch/#/]). In both, PC is the most abundant membrane lipid class (75 and 78%, respectively), followed by PE (18 and 17%), PI (5% and 3.5%), SL (1% and 0.6%) and PS (0.4% and 0.03%). The PS content of the deyolked embryo is significantly higher than in the yolk cell (Supplementary Fig. 1d–g). Within SL, dihydrosphingolipids (DHSL) as well as sphingomyelin (SM) with short-chain fatty acids are significantly less abundant in the yolk cell than in the deyolked embryo (for more details see Supplementary Fig. 1h–j).

### The levels of sphingolipids are regulated by Wnt signaling

**Fig. 1:**
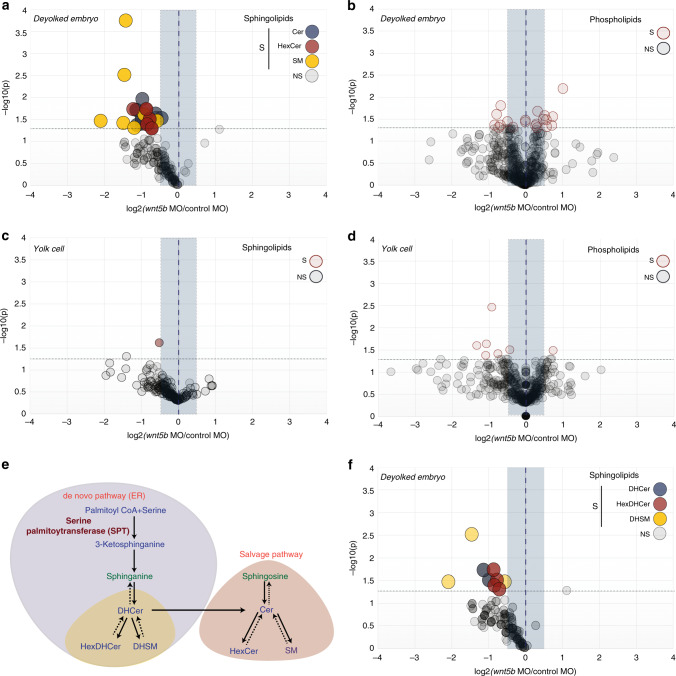
Wnt5b regulates sphingolipid levels in zebrafish embryos. **a**–**d**, **f** Volcano plots of lipidomic data for *wnt5b* morphants compared to control embryos at gastrula stage (6hpf at 28 °C) from four independent experiments. Horizontal dashed line thresholds significance (*p*-value = 0.05). NS (statistically non-significant); S (statistically significant). Unpaired, two-tailed student’s *t*-test was used. Blue shadowed area delimits +/−1.5-fold difference in abundance with respect to control. Relevant changes in the decrease of the content of a lipid species are located in the upper left quadrant. Each dot corresponds to an individual lipid species. **a**, **c** Sphingolipids in deyolked *wnt5b* morphant embryos (**a**) or in the yolk cell of *wnt5b* MO-injected embryos (**c**). Blue dots correspond to ceramides (Cer), red to Hexosylceramides (HexCer) and yellow to sphingomyelin (SM). **b**, **d** Phospholipids (PC, PE, PI, and PS) in deyolked *wnt5b* morphant embryos (**b**) or in the yolk cell of *wnt5b* MO-injected embryos (**d**). Note that the phospholipids significantly downregulated or upregulated beyond 1.5-fold in **b** and **d** correspond to phospholipids present at very low levels (between 1.3 x 10^−3^ and 8.4 x 10^−7^ Mol%) and therefore their amounts cannot be precisely determined in control versus *wnt5b* morphant conditions. **e** Simplified scheme representing both the de novo and salvage pathways for sphingolipid biosynthesis, with selected metabolites and genes indicated. DHCer dihydroceramide, HexDHCer dihydrohexosylceramide, DHSM dihydrosphingomyelin. **f** Volcano plot for sphingolipids in the de novo pathway in *wnt5b* deyolked morphant embryos. Lipid amounts are normalized as pmol/nmol inorganic phosphate (see “Methods”). Source data are provided as a Source Data file.

**Fig. 2:**
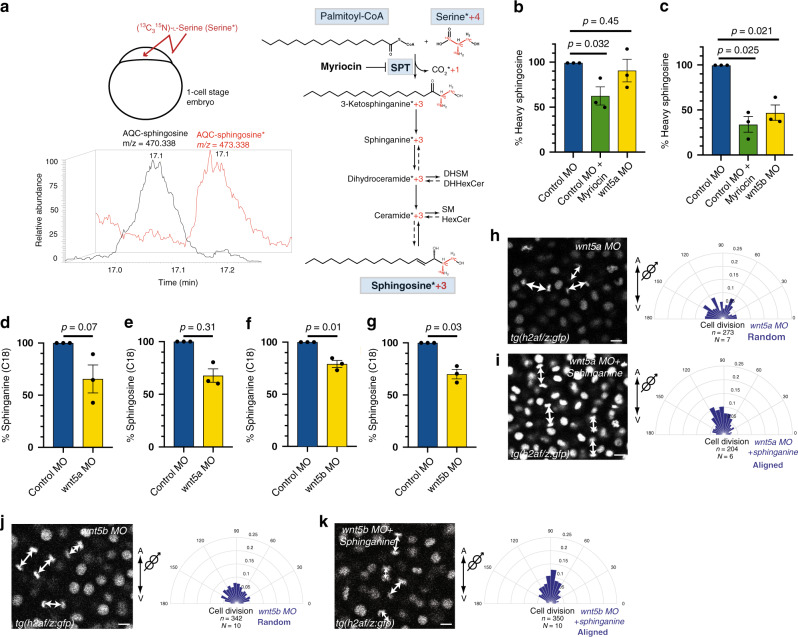
Wnt5b signaling regulates the levels of sphingolipids by modulating SPT activity. **a** Scheme of metabolic tracer assay. One-cell stage embryos were injected with (^13^C_3_^15^N)-l-serine (heavy serine/serine*), which is incorporated into 3-ketosphinganine by SPT, losing one heavy carbon. Incorporation of the remaining three heavy carbons into the downstream sphingoid base sphingosine is used as a direct in vivo readout for SPT activity. Heavy sphingosine (red) co-elutes with sphingosine (black), but it can be distinguished due to its higher mass/charge ratio (*m*/*z*). These experiments were done three times independently for each condition. **b**, **c** Relative amounts of heavy sphingosine in *wnt5a* morphant embryos (**b**), *wnt5b* morphants (**c**) or embryos treated with Myriocin (**b**, **c**) from three independent experiments. **d**, **e** Relative amounts of C18 sphinganine (**d**) or C18 sphingosine (**e**) in *wnt5a* morphant embryos from three independent experiments. **f**, **g** Relative amounts of C18 sphinganine (**f**) or C18 sphingosine (**g**) in *wnt5b* morphant embryos from three independent experiments. Unpaired, two-tailed student’s *t*-test was used in (**b**–**g**). All error bars represent the standard error of the mean (SEM). **h**–**k** Orientation of division of epiblast cells with respect to the A/V embryonic axis determined by using H2A.F/Z:GFP transgenic line to monitor chromosome separation during anaphase. Left, representative confocal images of dorsal epiblast cells expressing H2A-GFP in *wnt5a* morphant*s* (**h**), in *wnt5a* morphants treated with sphinganine (0.5 nl of 10 μM) (**i**), in *wnt5b* morphants (**j**) and in *wnt5b* morphants treated with sphinganine (0.5 nl of 25 μM) (**k**). Division axes are marked by arrows. Animal pole is up. Scale bars: 10 µm. Right, polar graphs showing the frequency distribution of the angle between the division axis and the A/V embryonic axis in *wnt5a* morphants (**h**) and in *wnt5a* morphants treated with sphinganine (0.5 nl of 10 μM) (**i**), *wnt5b* morphants (**j**) and in *wnt5b* morphants treated with sphinganine (0.5 nl of 25 μM) (**k**). *n* (number of cell divisions analyzed) over *N* (number of embryos). Note that rescue of randomization of division in *wnt5b* morphants requires higher amounts of sphinganine than for *wnt5a* morphants. In (**h**–**k**), *χ*^2^ test was used (see Supplementary Table 1). Source data are provided as a Source Data file.

Wnt5 proteins are non-canonical Wnt ligands that mediate oriented division of epiblast cells during zebrafish gastrulation^5^ (Supplementary Fig. 2a; see also Fig. 2h, j and Supplementary Movies 1 and 3). To identify membrane lipids involved in the regulation of oriented division of these cells, we used a validated *wnt5b* morpholino^24^ and analyzed the lipidome of gastrula stage embryos (Fig. 1). Deyolked *wnt5b* morphants show a decrease in the levels of sphingolipids compared to deyolked control morphant embryos (Fig. 1a and Supplementary Fig. 3i, j), while other membrane lipid classes (phospholipids) do not change significantly (Fig. 1b and Supplementary Fig. 3k). In contrast, the lipid profile of the yolk cell is mainly unchanged in *wnt5b* morphant embryos (Fig. 1c, d). Taken together, this indicates that (i) not only the yolk cell is metabolically active, but also the developing embryo at very early developmental stages has the ability to synthesize new lipids and (ii) the sphingolipid content in the embryo proper is controlled zygotically by Wnt signaling.

Sphingolipids are either derived from the recycling of other preexisting sphingolipids via the salvage pathway or from the de novo pathway via a common precursor, the sphingoid base sphinganine (Fig. 1e). The first sphingolipids generated by de novo synthesis are DHSL^25^ (yellow in Fig. 1e). The levels of DHSL are downregulated in *wnt5b* morphants (Fig. 1f and Supplementary Fig. 3i, j), indicating that Wnt signaling regulates the levels of sphingolipids generated through the de novo pathway.

### Wnt signaling modulates SPT activity

Serine palmitoyltransferase (SPT) catalyzes the first and rate-limiting step of the de novo sphingolipid pathway, which consists in the condensation of serine and palmitoyl-CoA to form 3-ketosphinganine. 3-ketosphinganine is rapidly reduced to sphinganine that is modified to produce ceramides and more complex sphingolipids, including sphingomyelin and hexosylceramides (HexCer)^19^ (Fig. 1e). Wnt signaling regulates the levels of sphingolipids produced through the de novo pathway (Fig. 1f and Supplementary Fig. 3i, j). Therefore, we next asked whether Wnt signaling regulates the levels of sphingolipids by modulating SPT activity (Fig. 2). To address this, we performed a heavy isotope serine tracer-based assay, which enabled us to unambiguously trace and quantify downstream metabolites, such as sphingosine, derived exclusively from the reaction catalyzed by SPT using heavy serine (Fig. 2a).

Using this approach, we tested whether both Wnt5 isoforms that are expressed at gastrula stages in zebrafish embryos (Wnt5a and Wnt5b)^26–28^ have an effect on SPT activity. One-cell stage embryos were treated with (^13^C_3_^15^N)-l-serine (heavy serine) and injected with either control MO, *wnt5a* MO, *wnt5b* MO or with Myriocin, a potent inhibitor of SPT^29^ that was used as a positive control. We found differences between Wnt5a and Wnt5b that seem to be merely quantitative: we found that Wnt5a also regulates the levels of sphingolipids although less efficiently than Wnt5b (Supplementary Fig. 3a–h). We observed that *wnt5a* knock down embryos showed very little effect on the levels of isotopically labeled sphingosine (Fig. 2b), raising the possibility that this function is redundant and can be also carried out by Wnt5b. Indeed, inhibition of Wnt5b results in a strong, significant reduction of heavy sphingosine levels (Fig. 2c), indicating that Wnt5b signaling does regulate sphingolipid levels by modulating the activity of SPT.

Consistent with a role of Wnt5 in modulating SPT activity, we also observed that both *wnt5a* and *wnt5b* morphant embryos showed a decrease in the levels of both sphinganine and sphingosine although only in *wnt5b* morphants this reduction is statistically significant (Fig. 2d–g and Supplementary Fig. 5a). Furthermore, while 0.5 nl of 10 μM sphinganine is sufficient to restore the orientation of division in epiblast cells in *wnt5a* morphant embryos (Fig. 2h, l and Supplementary Movies 1 and 2 and Supplementary Fig. 2b), at least 0.5 nl of 25 μM sphinganine is required to restore oriented division in *wnt5b* morphant embryos (Fig. 2j, k and Supplementary Movies 3 and 4). Taken together, these data show that Wnt5b regulates the levels of sphingolipids through SPT. In addition, Wnt5a has a lesser impact on sphingolipid regulation. We speculate that the Wnt5a morphant phenotype may be rather caused by its regulation of the actin cytoskeleton, as previously shown^5^.

### Sphingolipids are required for oriented divisions of epiblast cells

Wnt signaling thereby tunes the overall levels of sphingolipids available at the membrane for a plethora of cellular functions by regulating the activity of SPT. To test whether sphingolipids play a role during oriented divisions of epiblast cells, we interfered with the de novo sphingolipid biosynthetic pathway. SPT is a membrane protein complex^30–32^, which includes the SPTLC1 subunit essential for the enzymatic activity^31^. SPTLC1 is conserved in zebrafish (85% identity with the human SPTLC1), and *sptlc1* is ubiquitously transcribed during zebrafish gastrulation (Supplementary Fig. 4a).

**Fig. 3:**
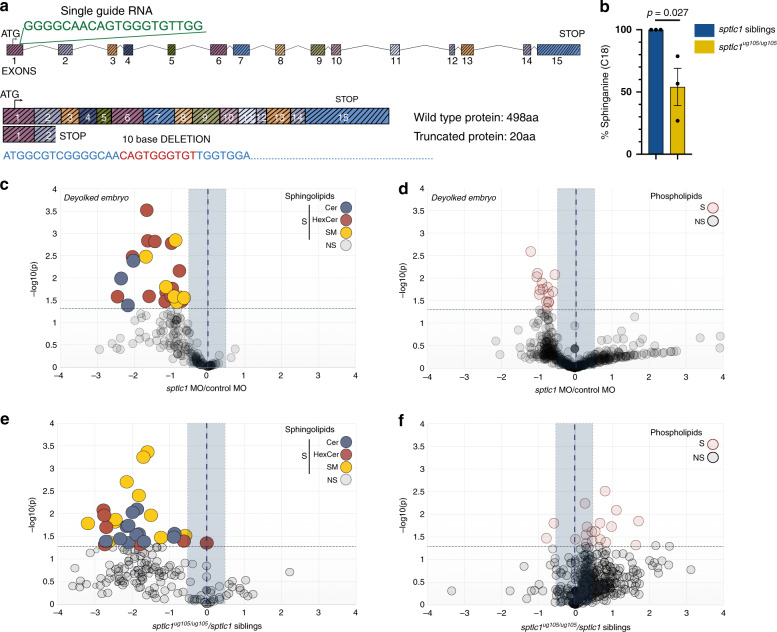
*sptlc1*-deficient embryos exhibit low levels of sphingolipids. **a** Structure of zebrafish *sptlc1* gene. Single-guide RNA used is shown in green. The sequence deleted in the CRISPR/Cas9 mutant is shown in red in the annotated *sptlc1* sequence. **b** Graph showing the relative amounts of C18 sphinganine in 4 dpf *sptlc1*^*ug105/ug105*^ normalized to the average of the amounts of C18 sphinganine in their siblings. Data are represented as the mean value +/- SEM from three independent experiments. Unpaired, two-tailed student’s *t*-test was used. **c**–**f** Volcano plots of lipidomic data from three (**c**, **d**) or two (**e**, **f**) independent experiments showing either sphingolipids in deyolked embryos for *sptlc1* morphants at gastrula stages (**c**) or in 4 dpf *sptlc1*^*ug105/ug105*^ mutants (**e**) or phospholipids in deyolked embryos for *sptlc1* morphants at gastrula stages (**d**) and 4 dpf *sptlc1*^*ug105/ug105*^ mutants (**f**). Horizontal dashed line thresholds significance (*p*-value = 0.05). NS (statistically non-significant); S (statistically significant). An unpaired, two-tailed student’s *t*-test was used for analysis of *sptlc1* morphants. A paired, two-tailed student’s *t*-test was used for *sptlc1* mutants. Blue shadowed area delimits +/–1.5-fold difference abundance with respect to control. Relevant changes in the decrease of the content of a lipid species are located in the upper left quadrant. Each dot corresponds to an individual lipid species. Note that phospholipids downregulated in deyolked *sptlc1* morphants and upregulated in *sptlc1*^*ug105/ug105*^ mutants correspond mainly to PE and minor PI species (for details see Supplementary Fig. 5e, h). Lipid amounts were normalized as pmol/nmol inorganic phosphate (see “Methods”). Source data are provided as a Source Data file.

To address whether sphingolipids are important for oriented division of epiblast cells, we generated *sptlc1* morphants and CRISPR mutants (Fig. 3a). The *sptlc1*^*ug105*^ mutant allele carries a deletion of 10 bases resulting in a premature stop codon in the middle of exon 2, generating a truncated protein of 20 amino acids (aa) compared to the 498 aa wild-type protein. The mutation therefore deletes most of the functional domains of the Sptlc1 protein (Fig. 3a). Homozygous mutants for *sptlc1*^*ug105*^ (*sptlc1*^*ug105/ug105*^) showed clear morphological defects at around 4 days post fertilization (dpf), that included small body size, curved trunk and lack of gas bladder inflation (Supplementary Fig. 4b). Homozygous mutants die at 5–6 dpf. At gastrula stages, these embryos are visually indistinguishable from their wild type and heterozygous siblings. Moreover, similar to embryos defective in non-canonical Wnt signaling^28,33^, both *sptlc1* mutants and morphants showed a shortening of the anterior-posterior axis of the embryo at the end of gastrulation (Supplementary Fig. 4d–g).

Sphingoid base analysis showed that the levels of sphinganine are indeed reduced around 50% in 4 dpf *sptlc1*^*ug105/ug105*^ mutant embryos (Fig. 3b). In addition, lipidomic analysis of both 4 dpf *sptlc1*^*ug105/ug105*^ mutant embryos or *sptlc1* morphants at gastrula stages showed that the levels of sphingolipids are strongly reduced (Fig. 3c, e and Supplementary Fig. 5b–d, f, g), while other lipid classes are less affected (Fig. 3d, f and Supplementary Fig. 5e, h).

**Fig. 4:**
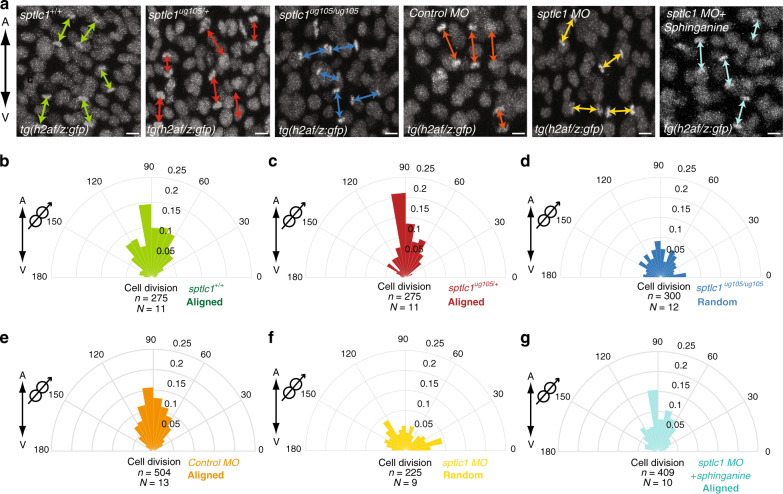
Sphingolipids regulate oriented divisions of epiblast cells. **a**–**g** Orientation of division of dorsal epiblast cells with respect to the A/V embryonic axis determined by using H2A.F/Z:GFP transgenic line to monitor chromosome separation during anaphase. **a** Representative confocal images of dorsal epiblast cells expressing H2A-GFP in *sptlc1*^*+/+*^, *sptlc1*^*ug105/+*^ and *sptlc1*^*ug105/ug105*^ embryos and in control, *sptlc1* morphants and *sptlc1* morphant embryos treated with sphinganine (from left to right) from at least nine independent experiments. Division axes are marked by arrows. Animal pole is up. Scale bars: 10 µm. **b**–**g** Polar graphs showing the frequency distribution of the angle between the division axis and the A/V embryonic axis from (**a**) in *sptlc1*^*+/+*^, (**b**) *sptlc1*^*ug105/+*^, (**c**) *sptlc1*^*ug105/ug105*^, (**d**) control morphants, (**e**) *sptlc1* morphants, (**f**) and *sptlc1* morphants treated with sphinganine (**g**). *n* (number of cell divisions analyzed) over *N* (number of embryos). Divisions are aligned along the A/V axis in *sptlc1*^*+/+*^, *sptlc1*^*ug105/+*^ and in control morphant embryos, but randomized in *sptlc1*^*ug105/ug105*^ embryos and *sptlc1* morphants. Randomization of division in *sptlc1* morphants is rescued by injection of 0.5 nl of 0.25 μM sphinganine. In (**b**–**g**), *χ*^2^ test was used (see Supplementary Table 1). Cartoons at the corner of polar graphs in this report indicate the parameter measured. Source data are provided as a Source Data file.

This reduction in sphingolipids resulted in impaired oriented epiblast cell division, since orientation is randomized both in *sptlc1*^*ug105/ug105*^ embryos and in *sptlc1* morphants (Fig. 4a–g, Supplementary Fig. 6a, b and Supplementary Movies 5–9). Consistently, sphinganine addition to the morphants restored the orientation of mitosis in epiblast cells (Fig. 4a, g, Supplementary Movie 10 and Supplementary Fig. 6c, d). This indicates that the reduced sphingolipid levels in Sptlc1 impaired embryos is what causes the observed defects in the orientation of mitosis and that sphingolipids are therefore essential during oriented division.

### Sphingolipids mediate Diaphanous-dependent rotation of the spindle

**Fig. 5:**
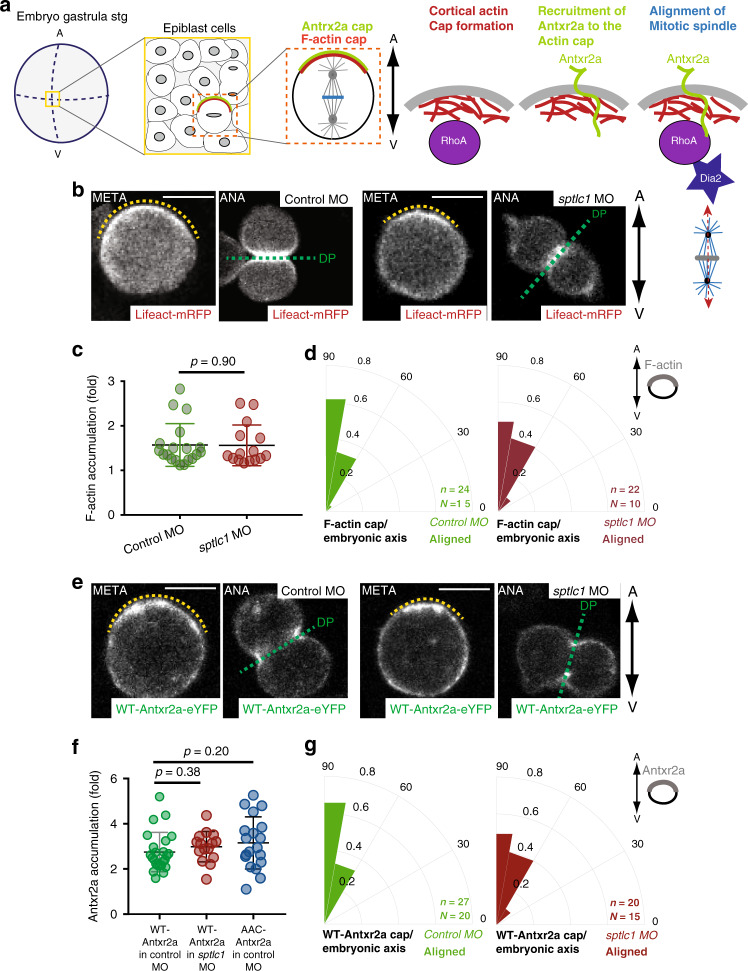
Formation of F-actin and Antxr2a caps are sphingolipid-independent. **a** Scheme representing the three functions of the actin cytoskeleton during oriented divisions of epiblast cells^5^. In metaphase, epiblast cells form a polarized F-actin cap aligned with respect to the A/V axis. The formation of this F-actin cap depends on the small GTPase RhoA. The transmembrane protein Antxr2a binds to actin, and it is therefore recruited to and enriched at the actin cap to form a cap itself. Both caps colocalize. Antxr2a interacts then with an active form of RhoA to activate Diaphanous, which connects the mitotic spindle with the caps, rotating the spindle to align it with the caps and therefore with the embryonic axis. As a result, divisions of epiblast cells are oriented along the A/V embryonic axis. **b**, **e** Representative confocal images from a time-lapse movie of epiblast cells at metaphase (META) and anaphase (ANA) expressing the F-actin biosensor **(**Lifeact-mRFP) (**b**) or Antxr2a-eYFP (**e**) in control embryos (left panels) or *sptlc1* morphants (right panels) from at least ten independent experiments. Yellow dashed line highlights F-actin or Antxr2a cap. Green dashed line, division plane. Animal pole, up. The embryonic axis is represented by a bi-directional arrow on the right. Scale bars: 10 µm. **c** Quantification of cortical F-actin accumulation at the cap of epiblast cells in control (*n* = 19 cells; green) and *sptlc1* morphants (*n* = 15 cells; red) expressing Lifeact-mRFP. **d**, **g** Polar graphs showing the frequency distribution of the angle between the F-actin cap (**d**) or the Antxr2a cap (**g**) and the plane of the A/V embryonic axis in control (left graph) and *sptcl1* morphants (right graph). *n* (number of cells analyzed) over *N* (number of embryos). In (**d**, **g**), *χ*^2^ test was used (see Supplementary Table 1). **f** Antxr2a accumulation at the cap in epiblast cells expressing WT-Antxr2a-eYFP in control (*n* = 24 cells; green) or in *sptlc1* morphants (*n* = 15 cells; red) and expressing AAC-zAntxr2a-eYFP in control morphants (*n* = 19 cells; blue). Data in (**c**, **f**) are represented as the mean value +/− SEM and an unpaired, two-tailed student’s *t*-test was used. DP (Division plane). Source data are provided as a Source Data file.

**Fig. 6:**
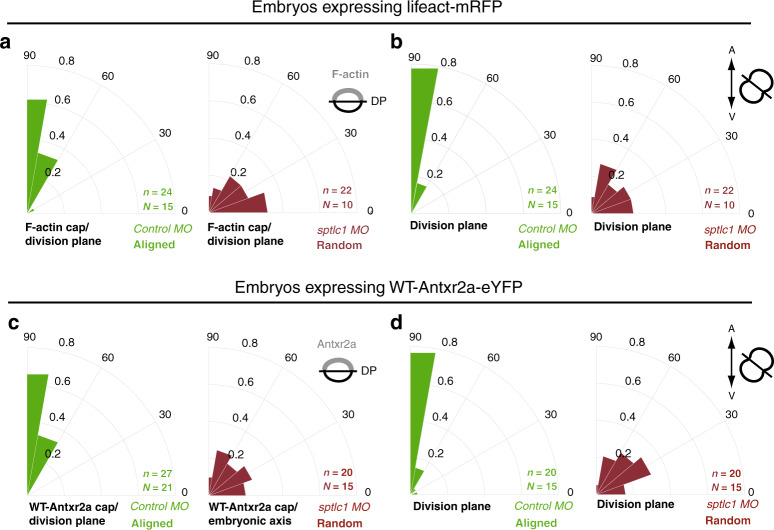
Alignment of F-actin and Antxr2a caps with the division plane requires sphingolipids. **a**, **c** Polar graphs showing the frequency distribution of the angle between the F-actin cap (**a**) or the Antxr2a cap (**c**) with respect to the mitotic plane (green line in Fig. 5b, e). **b**, **d** Polar graphs showing the frequency distribution of the angle between the plane of division and the plane of the embryonic axis in epiblast cells expressing either Lifeact-mRFP biosensor (**b**) or Antxr2a-eYFP (**d**) in control and *sptlc1* morphant embryos. *χ*^2^ test was used (see Supplementary Table 1). *n* (number of cells analyzed) over *N* (number of embryos). DP division plane. Source data are provided as a Source Data file.

Sphingolipids have been implicated in the dynamics of the actin cytoskeleton, which itself mediates a plethora of cellular processes such as endocytosis, exocytosis, vesicular trafficking and cell polarity^34^. We have previously shown that the actin cytoskeleton plays three distinct roles during oriented division of epiblast cells^5^: (i) in metaphase, the actin cytoskeleton is organized in a RhoA-dependent polarized cortical F-actin cap, which aligns with the embryonic axis, (ii) F-actin binds to Antxr2a, recruiting the receptor to the cap and, (iii) actin torques the mitotic spindle until it becomes aligned with the cap in a Diaphanous-dependent manner (Fig. 5a). By interfering with *sptlc1* function, we dissected which of these three steps is controlled by sphingolipids (Figs. 5 and 6).

Epiblast cells in *sptlc1* morphants form a normal polarized F-actin cap (Fig. 5b, c), which is aligned with respect to the embryonic axis (Fig. 5d). Sphingolipids are also not involved in Antxr2a recruitment to the F-actin cap, since the Antxr2a cap forms normally (Fig. 5e, f) and is aligned with the embryonic axis in *sptlc1* morphants (Fig. 5g).

However, in *sptlc1* morphant embryos, the mitotic spindle failed to rotate to align with respect to the F-actin and the Antxr2a caps (Fig. 6a, c), resulting in randomized orientation of divisions (Fig. 6b, d).

These data indicate that sphingolipids are not implicated neither in the formation or polarization of the caps nor in their alignment with the embryonic axis. Sphingolipids are, however, required to mediate the activity of these caps to regulate the last step, the Diaphanous-dependent rotation of the spindle, so that the mitotic plane is aligned with the caps and therefore properly oriented with the embryonic axis.

### Sphingolipids regulate Antxr2a palmitoylation

We have previously shown that the Diaphanous-dependent rotation of the mitotic spindle is mediated by the Anthrax toxin receptor itself, which interacts with RhoA to regulate Diaphanous activity^5^ (Fig. 5a). Therefore, we first looked at the effects of decreasing the levels of sphingolipids on the Anthrax toxin receptor itself.

Sphingolipids interact with palmitoylated proteins to determine their localization, trafficking, activity, and stability as well as the interactions with other proteins^35,36^. S-palmitoylation is a reversible lipid modification that consists in the enzymatic attachment of a 16-carbon fatty acid (usually palmitate C16:0) to a cysteine residue of a protein^37^.

**Fig. 7:**
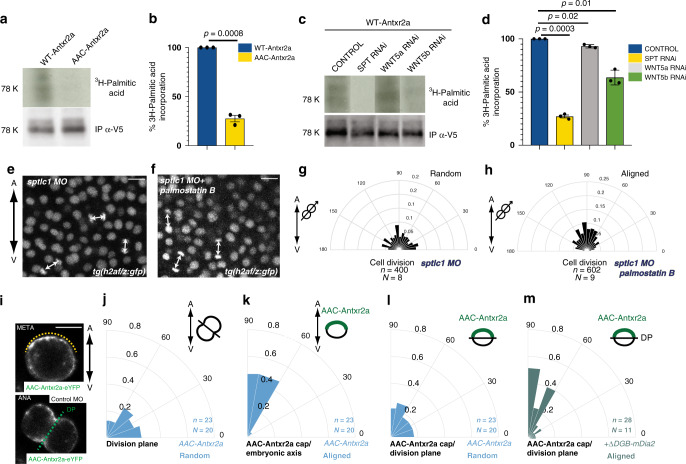
Sphingolipids regulate Antxr2a palmitoylation to control oriented divisions. **a**, **c** ^3^H-palmitate incorporation assay. Representative blots showing ^3^H-palmitate levels incorporated into WT-zAntxr2 or AAC-zAntxr2a expressed in HeLa cells (**a**) or into WT-zAntxr2a expressed in either Control, SPT, WNT5a or WNT5b RNAi HeLa cells (**c**). **b**, **d** Quantification of ^3^H-palmitate incorporation as shown in (**a**, **c**), normalized to the average of incorporation in WT-zAntxr2a from three independent experiments. Silencing SPT or WNT5b results in a reduction of Antxr2a palmitoylation levels. WNT5a silencing causes a mild, but statistically significant effect on receptor palmitoylation. Data are represented as the mean value +/– SEM. Unpaired, two-tailed student’s *t*-test was used. **e**–**h** Division orientation of epiblast cells with respect to the A/V embryonic axis. **e**, **f** Representative confocal images of epiblast cells expressing H2A-GFP in *sptlc1* morphants (**e**) or in *sptlc1* morphants treated with palmostatin B (**f**) from at least eight independent experiments. Division axes are marked by arrows. Animal pole is up. Scale bars: 10 µm. **g**, **h** Polar graphs showing the frequency distribution of the angle between the division axis and the A/V axis in *sptlc1* morphants and *sptlc1* morphants treated with palmostatin B. *χ*^2^ test was used (see Supplementary Table 1). **i** Representative confocal images from a time-lapse movie of a dorsal epiblast cell at metaphase (META) and anaphase (ANA) expressing AAC-Antxr2a-eYFP in control morpholino-injected embryos. Yellow dashed line corresponds to AAC-Antxr2a cap. Green dashed line, division plane (DP). Embryos are oriented with the animal pole up. Embryonic axis is represented by a bi-directional arrow on the right. Scale bars: 10 µm. **j**–**m** Polar graphs showing the frequency distribution of either the angle between the division plane (green dashed line in **i**) and the A/V embryonic plane (**j**), the angle between the Antxr2a cap and the A/V embryonic axis (**k**) or the angle between the cap and the division plane of epiblast cells in live embryos expressing AAC-Antxr2a-eYFP (**l**) or expressing AAC-Antxr2a and ΔBDG-mDia2 (**m**). *χ*^2^ test was used (see Supplementary Table 1). *n* (number of cells analyzed) over *N* (number of embryos). Source data are provided as a Source Data file.

The two human Anthrax toxin receptors, Antxr1/TEM8 and Antxr2/CMG2, are palmitoylated^8^. Antxr2/CMG2 and the zebrafish Antxr2a have three cytoplasmic cysteine residues that are conserved and palmitoylated in Antxr1/TEM8, two of which are juxta-membranous (Supplementary Fig. 7a). Figure 7a shows that zebrafish Antxr2a is indeed palmitoylated, as revealed by ^3^H-palmitate incorporation experiments in HeLa cells: a mutated form of Antxr2a, where the two juxta-membrane cysteines are substituted by alanine (AAC-Antxr2a), showed a significant decrease in the levels of ^3^H-palmitate incorporation compared to WT-Antrx2a (Fig. 7a, b).

We used this assay to study whether sphingolipids are required for Antxr2a palmitoylation. We found that upon SPT knock down, the levels of Antxr2a palmitoylation are strongly reduced (Fig. 7c, d and Supplementary Fig. 8i). Consistent with this, we found that downregulation of Wnt5b, which efficiently modulates SPT activity (Fig. 2c), results in a significant reduction in Antxr2a palmitoylation, while downregulation of Wnt5a results in a much weaker reduction (Fig. 7c, d).

S-palmitoylation is regulated by two types of enzymes, the palmitoyl acyltransferases (PAT) that add the fatty acid, and the acyl protein thioesterases (APT) that remove it^38^. We find that sphingolipids mediate Antxr2a palmitoylation by regulating APTs: in *sptlc1* morphant embryos treated with the APT inhibitor palmostatin B, the alignment of the mitotic spindle is restored (Fig. 7e–h, Supplementary Movie 11 and Supplementary Fig. 7e–g).

### Antxr2a palmitoylation is required for oriented divisions

The observation that sphingolipids regulate the palmitoylation state of Antxr2a opened the possibility that the division defects seen in *sptlc1* mutants and morphants are mediated by a decrease in the levels of Antxr2a palmitoylation. To address this, we expressed AAC-Antxr2a-eYFP in control MO-injected embryos. We found that AAC-Antxr2a expressed in control morphant embryos behaves like WT-Antxr2a expressed in *sptlc1* MO-injected embryos: expression of AAC-Antxr2a resulted in randomization of epiblast cell division orientation (Fig. 7i, j). This randomization, in contrast to what we found in *sptlc1* and *wnt5b* morphants, cannot be rescued by adding sphinganine (Supplementary Fig. 7b–d), indicating that palmitoylation is downstream of sphingolipids.

The palmitoylation state of Antxr2a does not affect neither its mobility in the plasma membrane nor its half-life (Supplementary Fig. 8a–d). Likewise, Antxr2a palmitoylation does not affect neither the sorting of the receptor nor the recruitment of the receptor to the F-actin cap (Figs. 5f and 7i): AAC-Antxr2a-eYFP expressed in MO-injected embryos formed a normal cap that is aligned with respect to the embryonic axis (Fig. 7k).

### Antxr2a palmitoylation determines spindle rotation by Dia

The defects observed upon sphingolipid depletion when Sptlc1 is impaired (Figs. 5 and 6) are also the same as those observed when the actin regulator Diaphanous is compromised: the Antxr2a and F-actin caps are formed and aligned with the embryonic axis, but they fail to torque the mitotic spindle. Therefore, the division plane is neither aligned with the caps nor with the embryonic axis^5^. This implies that normal levels of sphingolipids are essential for Diaphanous activity. We also found that normal levels of sphingolipids are necessary for Antxr2a palmitoylation (Fig. 7c), raising the possibility that lipid modification of the receptor is required for Diaphanous activity.

We first analyzed whether Antxr2a palmitoylation is required for the interaction of the receptor with RhoA or with its downstream effector Diaphanous itself. We found that, in both HeLa cells and in zebrafish embryos, interaction of RhoA, GTP-RhoA or Dia with Antxr2a is not impaired neither upon knock down of SPT, nor upon mutation of the juxta-membrane cysteines (Supplementary Fig. 8g, h). This indicates that, not Diaphanus binding, but Diaphanous activity per se is compromised when the receptor is not palmitoylated at juxta-membrane positions. This explains that expression of palmitoylation defective Anthrax toxin receptor recapitulates the torqueing defects seen in both *diaphanous* and *sptlc1* morphants: like WT-Antxr2a expressed in *dia* or *sptlc1* MO-injected embryos^5^ (Figs. 5f, g and 6c, d), AAC-Antxr2a expressed in control MO-injected embryos also formed a polarized cap (Figs. 5f and 7i), which was aligned with the embryonic axis (Fig. 7k), but failed to torque the spindle (Fig. 7l).

If palmitoylation of Antxr2a is indeed required for Diaphanous activity, then expression of a constitutively active Diaphanous protein should rescue the phenotype seen when receptor palmitoylation is defective. Indeed, the torqueing defects of AAC-Antxr2a can be rescued when epiblast cells expressing AAC-Antxr2a were provided with a form of Diaphanous (ΔBDG-mDia2), which lacks the domain that binds the RhoA GTPase site. RhoA binding is known to prevent Diaphanous auto-inhibition and this Diaphanous mutant keeps Diaphanous in its active state^39^ (Fig. 7m). This prompts the speculation that juxta-membrane palmitoylation of the receptor may change the three-dimensional (3D) conformation of its cytosolic tale to facilitate the presentation of bound Diaphanous to RhoA for efficient Diaphanous activation.

## Discussion

We have shown that the orientation of cell division is controlled by sphingolipids. Wnt signaling fine tunes the levels of sphingolipids in the cell by modulating the activity of SPT, the enzyme that catalyzes the first and rate-limiting step in de novo sphingolipid biosynthesis. Sphingolipids control the palmitoylation of Antxr2a, which we have previously shown to be required for oriented division. Epistasis experiments showed that sphingolipids regulate the palmitoylation state of Antxr2a by regulating APTs, the enzymes that remove the palmitate group from the cysteines. Palmitoylation of the juxta-membrane cysteines in Antxr2a enables the activation of Diaphanous bound to the receptor, which is essential to rotate and align the spindle with the caps and therefore with the embryonic axis.

These findings are based on the following key observations: (i) lipidomics and metabolic tracer analyses revealed that downregulation of Wnt signaling results in a decrease in the levels of sphingolipids in the embryo, including sphingolipids that are zygotically synthesized de novo (Figs. 1 and 2 and Supplementary Fig. 3); (ii) isotope tracer-based metabolomics showed that Wnt signaling regulates sphingolipids by modulating the activity of SPT (Fig. 2); (iii) interfering with the sphingolipid biosynthesis (*sptlc1* mutant or morphants) resulted in randomized epiblast cell divisions (Figs. 3 and 4); (iv) a reduction in the levels of sphingolipids results in a decrease in Antxr2a palmitoylation levels (Fig. 7); (v) decreasing the levels of sphingolipids or expressing a palmitoylation defective Antxr2a resulted in randomized divisions without interfering with the formation and alignment of the polarized F-actin and Antxr2a caps (Figs. 5–7). This phenotype is reminiscent of the one observed in *diaphanous* impaired embryos; (vi) epistasis experiments showed that the mitotic spindle rotation defects seen in AAC-Antxr2a-expressing cells are reverted by expressing a constitutively active form of Diaphanous (Fig. 7m). These data reveal a key role of the membrane lipid composition to control oriented cell division and uncover the mechanism by which Wnt/PCP can control oriented mitosis. We show how sphingolipids can regulate a developmental process under the control of signaling molecules.

In this scenario, at the polarized caps, activation of Diaphanous by RhoA mediates the torque of the spindle. This requires not only that Antxr2a is present at the cap, but it also requires that the receptor is palmitoylated. The state of palmitoylation of Antxr2a is determined by Wnt signaling that controls the levels of sphingolipids, which in turn controls APTs. This provides the cell with multiple layers of control by the Wnt signaling pathway to achieve a precise torqueing and alignment of the spindle: (i) Wnt/PCP entrains the orientation of an F-actin cap that otherwise can self-organize randomly in the absence of Wnt signaling, however, (ii) Wnt/PCP signaling is also required to control the levels of sphingolipids and thereby the palmitoylation of Antxr2a, functioning as a switch to control Diaphanous activity. In this system, mitotic spindle torqueing happens only when both the Antxr2a is recruited to the F-actin cap and the Wnt signaling regulates the levels of sphingolipids. This would ensure that planar cell polarity regulated by Wnt signaling instructs when and in which orientation cell division takes place.

## Methods

### Generation of *sptlc1*^*ug105*^ mutant embryos

*sptlc1*^*ug105*^ mutant embryos were generated by using the CRISPR/Cas9 genome editing technology. ZiFiT (zinc finger targeter) web-based tool was used to find the appropriate target sequence to be mutated. The following sgRNA was then selected: 5′-GGGGCAACAGTGGGTGTTGG-3′, where the PAM sequence is underlined. To create the *sptlc1* sgRNA, the following oligonucleotides with overhanging ends for *Bsa*I were designed: 5′-TAGGGGCAACAGTGGGTGTTGG-3′ and 5′-AAACCCAACACCCACTGTTGCC-3′ (Microsynth). 10 μl of each oligonucleotides (100 μM) were denatured at 95 °C for 5 min and annealed by cooling to 50 °C using a 0.5 °C/30 s ramp and then cloned into *Bsa*I-HF-digested pDR274 vector (Addgene). The plasmid was then linearized using *Dra* I restriction enzyme and transcribed using the Megashortscript T7 kit (Invitrogen, AM1354). For capped *cas9* messenger RNA (mRNA) synthesis, pT3TS-nCas9n plasmid was linearized by *Xba* I restriction digestion and transcribed by T3 polymerase using the mMessage mMachine Kit (Ambion). A mix of 12.5 ng/μl of *sptlc1* sgRNA and 300 ng/μl of *cas9* mRNA were injected in zebrafish wild type line AB embryos at 1-cell stage. Mutation efficiency was about 80%, most of the mutations resulting in three base pair (bp) deletions. Mutants were identified by amplifying a 300 bp region using the following primers: Forward primer (G-sptlc1-Forward): 5′-GCAGCCTATATTAAATGTTC-3′; Reverse primer (G-sptlc1-Reverse): 5′-ACAGCCGAATACAACCACCT-3′. Mutant fish used in this report consisted of a 10 bp deletion just after the ATG start codon, resulting in a premature stop codon at the end of the second exon. Identified mutant fish were outcrossed with wild-type AB and wild-type TL fish for four generations and kept as heterozygous mutant fish. Homozygous mutants for *sptlc1*^*ug105*^ (*sptlc1*^*ug105/ug105*^) were obtained from crossing the F4 heterozygote animals to minimize off-target effects. *sptlc1*^*ug105*^ heterozygous mutants were crossed to obtain *sptlc1*^*+/+*^, *sptlc1*^*ug105/+*^, and *sptlc1*^*ug105*/*ug105*^ littermates for phenotypic analyses.

### Staging and maintenance of embryos

This study followed European Union directives (2010/63/EU), the Swiss Animal Protection Act and the Swiss Animal Welfare Ordinance. Zebrafish embryos were staged according to ref. ^40^, grown at 30 °C and manipulated in Danieau media. Wild-type line AB was used for injection of different morpholinos for the lipidomic and metabolic experiments. To address the orientation of the division of epiblast cells, the Tg(*h2afva:h2afva-GFP*) line was used. *sptlc1*^*ug105/+*^ heterozygous mutants were crossed to Tg(*h2afva:h2afva-GFP*) to create an *sptlc1*^*ug105/+*^;Tg(*h2afva:h2afva-GFP*) stable line.

### Plasmids, chemicals, mRNA, and morpholino injections

pCS2-WT-Antxr2a-eYFP and pCS2-Lifeact-mRFP plasmids were used for zebrafish embryo injections^5^. pCS2-WT-Antxr2a-eYFP was used to generate the pCS2-AAC-Antxr2a-eYFP using the QuikChange Site-directed kit from Stratagene (La Jolla, CA) according to the manufacture’s protocol with the following primers: Forward primer (AAC-Forward): 5′-GGTCTTTAATAACGACAGTGGCGGCTAGAGGCCAGAACCACCACATCA-3′; and Reverse primer (AAC-Reverse): 5′-TGATGTGGTGGTTCTGGCCTCTAGCCGCCACTGTCGTTATTAAAGACC-3′.

Zebrafish Sptlc1 complementary DNA (cDNA) was obtained from Dharmacon (Catalog number: MDR1734-202793948). This cDNA was cloned into pCS2+ vector using the following primers:

Forward primer (sptlc1-Forward): 5′-CGGAATTCATGGCGTCGGGGCAACAGTG-3′; and Reverse primer (sptlc1-Reverse): 5′-CCGCTCGAGTCATTTCAGGACGTGTAAAG-3′ containing an *Eco* RI and *Xho* I restriction sites.

Three non-overlapping morpholinos, two against the 5′-UTR sequence of the zebrafish *sptlc1* gene (sptlc1-1-UTR MO: 5′-GCGACTTCCTGATTGATAAAGAGGC-3′; sptlc1-2-UTR MO: 5′-CGCCATTTTGACTGAGTCACATGAT-3′, and another against the coding sequence (sptlc1-ATG MO: 5′-ACCCACTGTTGCCCCGACGCCATTT-3′) (Gene Tools, Inc.) were used to knock down *sptlc1* in zebrafish embryos. As a control morpholino, we used a 3-nucleotide mismatch of the ATG MO. Experiments were carried out by injecting 1-cell stage, wild-type AB line embryos using either 2 ng of one of the two *sptlc1* UTR MO, 0.5 ng of the ATG MO or 0.5 ng of the control MO. To knock down either *wnt5a* or *wnt5b*, 2 ng of *wnt5a* MO (5′-TCCACTTCAGCTTCAGCAGCATCAT-3′)^5,41^ or 4 ng of *wnt5b* MO (5′-GTCCTTGGTTCATTCTCACATCCAT-3′)^24^ were used.

To visualize F-actin, Antxr2a or AAC-Antxr2a, either 25 pg of the biosensor *lifeact-mRFP* or 75 pg of *antxr2a-eYFP or* AAC-Antxr2a-eYFP mRNAs were injected into one cell of 16-cell stage, wild type or *sptlc1* (ATG MO) morphant embryos. F-actin, Antxr2a or AAC-Antxr2a cap formation was monitored by 3D confocal imaging in embryos at shield stage (6hpf at 28 °C).

The sphingoid base sphinganine was obtained from Avanti Polar Lipids (Alabaster, Alabama, USA). Ten micromolar stock solution was prepared in ethanol. For the rescue experiment of oriented cell division, 0.5 nl of a 1:40 dilution was injected into *sptlc1* or *wnt5b* morphant embryos or in embryos expressing AAC-Antxr2a-eYFP.

The palmostatin B was obtained from Sigma-Aldrich (Reference #178501). Tg(*h2afva:h2afva-GFP*) embryos injected with *sptlc1* MO were developed until blastula stage. Then, embryos were incubated with 100 μM for 4 h, dechorionated by hand and imaged as described below. For the measurements of the length of the body axis in *sptlc1* morphant embryos treated with palmostatin B, the fish medium was exchanged several times after 4 h incubation with palmostatin B to remove any remaining compound.

For the co-localization experiments of Antxr2a to recycling endosomes, 100 pg of *eCFP-Rab11* mRNA was injected together with 75 pg of *antxr2a-eYFP* into one-cell stage, wild-type embryos.

For the spindle rotation experiments, 50 pg *GFP-doublecortin* (*GFP-dcx*) mRNA was injected in one cell out of eight for mosaic expression in embryos injected with either *wnt5b* MO or *sptlc1* MO and treated or not with sphinganine as describe before.

For rescue of AAC-Antxr2a by Diaphanous expression, a constitutively active form of mDiaphanous, where the GTPase binding domain was deleted (pEFm-DGBD-mDia2) was used^39^, gift from Danijela Matic Vignjevic.

For capped mRNA synthesis, all the constructs were linearized by *Not* I restriction digestion and transcribed by SP6 polymerase using the mMessage mMachine Kit (Ambion).

### Detection of episodes of direct motion

To determine which parts (if any) of the mitotic spindle trajectory exhibited directed rotation, epiblast cells from embryos injected with *GFP-dcx* mRNA were imaged. Embryos were always oriented with the animal pole up. The angle of the mitotic spindle with respect the embryonic axis was calculating by measuring in ImageJ (version 2.0.0) the position for each centrosome over time. Using the same approach as in ref. ^5^, we calculated the *dai* (*directional auto-correlation index*) for each individual trajectory for a time window *L* = 4. The directional auto-correlation index allows us to determine whether the mitotic spindle of a cell undergoes transient directed rotation or whether it moves by random fluctuations^5^. For all the trajectories shown here, we have located clusters of dai = 1 and selected the clusters which have a size of at least four time points. These clusters correspond to directed episodes^5^.

### RNA in situ hybridization

Whole-mount in situ staining of zebrafish embryos was performed as previously described^42^. RNA in situ probes were synthesized from *no tail* (*ntl*)^43^, *distalex3* (*dlx3*)^44^ and *hatching gland gene-1* (*hgg*)^45^ cDNAs using a DIG RNA labeling kit (Roche). For *sptlc1* in situs, an antisense RNA probe was synthesized by PCR from a pCS2-Sptlc1 plasmid by using the following primers: forward primer (I-sptlc1-Forward): 5′- ATTGAAGATCAGAAGAATCCGCGTA-3′; and reverse primer (I-sptlc1-Reverse): 5′- **AATTTAATACGACTCACTATAGG**CGAGCCAACGTTAGTGCGACCTGTC-3′, where the T7 promoter has been included in the reverse primer (in bold). Antisense RNA was then synthesized using T7 RNA polymerase.

### ^3^H-palmitate incorporation

For the ^3^H-palmitate incorporation, HeLa cells were transfected with either WT-Antxr2a-V5 or AAC-Antxr2a-V5 for 48 h using FuGENE according to manufacturer’s protocol (Promega).

To address the palmitoylation state of WT-Antxr2a in different knock down conditions, HeLa cells were first transfected with 100 nM control Small interfering RNA (siRNA; (5′-ATTGAACAAACGAAACAAGGA-3′ targeting the viral glycoprotein VSV-G)), 100 nM WNT5a siRNA (5′-CCGGATAACCTTGTAACATAT-3′), 100 nM WNT5b siRNA (5′-GTTTATCATCGGTGCCCAGCC-3′) or 100 nM of both SPTLC1 and SPTLC2 (SPT) siRNAs (5′-CAAGAACGATCTGATCTTACA-3′; 5′-AAGGAGGTTGTGGTAACTAAA-3′), using INTERFERin (PolyPlus Transfection). After 24 h, these cells were also transfected with WT-Antxr2a-V5, using FuGENE. Cells were analyzed at least 72 h after siRNA transfection.

For ^3^H-palmitate incorporation, transfected cells were then incubated for 2 h at 37 °C in IM with 200 µCi/ml 3H-palmitic acid (9,10-3H(N); American Radiolabeled Chemicals, Inc). Posteriorly, cells were washed and immunoprecipitated with V5 monoclonal antibodies. For immunoprecipitation of the Antxr2a, cells were lysed for 30 min at 4 °C in immunoprecipitation buffer (0.5% NP-40, 500 mM Tris-HCl, pH 7.4, 20 mM EDTA, 10 mM NaF, 2 mM benzamide,1 mM N-ethyl-maleimide, and a cocktail of protease inhibitors), centrifuged for 3 min at 2.000 x *g* and supernatants were incubated overnight at 4 °C with protein G-coupled beads (GE Healthcare) with 2 μg monoclonal antibody against V5 (Thermofisher #R960, RRID:AB_2556564). After washing of the beads, samples were boiled for 5 min under reducing conditions prior to 4–20% gradient sodium dodecyl sulfate–polyacrylamide gel electrophoresis (SDS-PAGE). Gels are incubated 30 min in a fixative solution (25% isopropanol, 65% H_2_O, 10% acetic acid), followed by a 30 min incubation with signal enhancer Amplify NAMP100 (GE Healthcare). The radiolabeled products were revealed using Typhoon phosphoimager. The images shown for ^3^H-palmitate labeling were obtained using fluorography on film.

### Western blot and immunoprecipitation

For Immunoprecipitation and western blots carried out using zebrafish embryos, >100 wild type or *sptlc1* morphant embryos were injected with either 0.1 μg/μl *wt-antxr2a-eYFP* or 0.1 μg/μl *aac-antxr2a-eYFP* mRNA. Embryos were developed at 28 °C until they reached shield stage (6 hpf), dechorionated with 2 mg/ml Pronase (Roche applied Science), which was previously heated at 37 °C. Since dechorionated embryos younger than tailbud stage adhere to plastic, this step was performed in glass beakers. Embryos were treated with Pronase for 20 min and washed several times with fish medium.

Embryos were transferred from the glass beakers to a 1.5 ml tube filled with 1 ml deyolking buffer (1/2 Ginzburg Fish Ringer without Calcium: 55 mM NaCl, 1.8 mM KCl, 1.25 mM NaHCO_3_) by pipetting with a narrow tip so that the yolk sac is disrupted. Embryos were shaken for 5 min at 1100 r.p.m. to dissolve the yolk. Cells were pelleted at 300 x *g* for 30 s and the supernatant was carefully discarded. Two additionally wash steps were performed by adding 1 ml of wash buffer (110 mM NaCl, 3.5 mM KCl, 2.7 mM CaCl_2_, 10 mM Tris/Cl pH 8.5), shaking 2 min at 1100 r.p.m. and pelleting the cells as before. The excess of wash buffer was eliminated, and cells (deyolked embryos) were frozen in liquid nitrogen and stored at –80 °C^46^.

For immunoprecipitations, cells were washed with cold PBS, pelleted at 25 x *g*, 4 °C for 10 min, twice. Then, cells were transferred into a pre-cooled 1.5 ml tube and were lysed in 500 μl of ice-cold lysis buffer (25 mM NaF, 1 mM Na_3_VO_4_, 50 mM Tris pH 7.5, 1.5 mM MgCl_2_, 125 mM NaCl, 0.2% IGEPAL, 5% glycerol, 1 mM DTT, 1 tablet of cocktail of protease inhibitors (Roche)), leaving them on ice for 20 min. Lysates were pelleted at 400 x *g*, 4 °C for 10 min and the supernatant was transferred into a fresh tube kept on ice. Protein concentration was measured using the Bradford method. Obtained protein concentration was ~1.75 μg/μl.

HeLa cells expressing WT-Antxr2a or AAC-Antxr2a were incubated with 500 ng/ml Anthrax protective antigen PA (purified in the lab) at 4 °C for 1 h and switched at 37 °C for 10 min before lysis. Cells or embryo extracts were lysed 30 min at 4 °C in immunoprecipitation buffer (0.5% NP-40, 500 mM Tris-HCl, pH 7.4, 20 mM EDTA, 10 mM NaF, 2 mM benzamide,1 mM N-ethyl-maleimide, and a cocktail of protease inhibitors), centrifuged for 3 min at 2.000 × *g* and supernatants were incubated overnight at 4 °C with protein G-coupled beads (GE Healthcare) with 2 μg rabbit antibody against GFP (Clontech #632592, RRID: AB_2336883) or with 2 μg normal rabbit IgG as a control (Santa Cruz, sc-2027). After washing of the beads, samples were boiled for 5 min under reducing conditions prior to 4–20% gradient SDS-PAGE. Western blots were revealed with mouse anti-GFP (Sigma, #11814460001, RRID: AB_390913; dilution 1:500), rabbit anti-rhoA (Cell signaling, #2117, RRID: AB_10693922; dilution 1:2000), rabbit anti-Dia (Thermofisher, # PA5-21409, RRID: AB_11153295; dilution 1:1000), V5 antibody (Thermofisher #R960, RRID:AB_2556564; dilution 1:2000), rabbit anti-zAntxr2a (EUROGENTEC; H2N-NKG LKT LSE VNP AGE C-CONH2); dilution 1:500) and mouse anti-active RhoA (NEWEAST 26904 RRID:AB_1961799; dilution 1:1000).

To address the deyolking efficiency, western blots were performed as described above using either 50 intact embryos or 50 deyolked embryos. Embryos were lysed with 60 µl of ice-cold 1x lysis buffer (25 mM NaF, 1 mM Na_3_Vo_4_, 50 mM Tris-HCL pH 7.5, 1.5 mM MgCl_2_, 125 mM NaCl, 0.2% IGEPAL, 5% glycerol, cOmplete^TM^ mini EDTA-free protease inhibitor cocktail (Merck, 11836153001). Lysates were pelleted at 16,000 x *g*, 4 °C for 10 min and the supernatant was transferred into a fresh tube kept on ice. Samples were diluted in NuPAGE LDS sample buffer + 10% β-mercaptoethanol, boiled for 5 min at 100 °C and loaded in 12% NuPAGE Bis-Tris Gel. After dry blotting and blocking with 5% milk, western blots were revealed using a 1:500 dilution of the anti-Vitellogenin monoclonal antibody (Cat# DMABT-Z60323, Creative Diagnostics). To quantify bands on SDS-PAGE, Gels script in ImageJ software was used.

### Embryo mounting and confocal imaging

Time lapses to visualize either the Antxr2a cap, F-actin cap or cell division orientation were done in embryos at shield stage that were manually dechorionated and mounted in 1% low-melting-point agarose in Danieau fish medium. To measure oriented division of *sptlc1* mutant embryos, *sptlc1*^*ug105/+*^;tg (*h2afva:h2afva-GFP*) siblings were crossed and the embryos collected. Two or three movies per cross were imaged. After imaging, to correlate the phenotype observed in the orientation of divisions of epiblast cells with the genotype of the embryo, the embryos were removed from 1% low-melting-point agarose, their genomic DNA extracted, a DNA fragment of about 300 bp around the mutation amplified by PCR and sequenced.

Four-dimensional (4D) confocal time-lapse imaging of dorsal epiblast cells was performed at room temperature using an upright Zeiss 710 Multiphoton Confocal Microscope with a W-Plan-Apochromat 20×/1.0DIC M 27 70 mm lens. For Antxr2a and F-actin cap visualization, between 17 and 30 time points were acquired of defined *z-*stacks with a step size of 1 μm over a time interval of 1.2–1.5 min (depending on the size of the *z*-stack). For cell division orientation *z*-stacks were collected at 2 min intervals. Embryos were imaged for ~2–3 h.

### Image analysis and quantification

Confocal images were analyzed and quantified using Image-J [http://rbs.info.nih.gov/ij/]. For cell divisions, embryos were mounted so that the A/V axis corresponds to the *y*-axis. For each division, *x*,*y* coordinates were measured and the angle between the division axis and the A/V axis of the embryo calculated. In polar graphs, an angle of 90° corresponds to cells that divide parallel to the *y-*axis (along the A/V axis) and an angle of 0° corresponds to cells that divide perpendicular to the *y-*axis (perpendicular to the A/V axis).

To calculate the F-actin accumulation at the cap, the Lifeact-mRFP biosensor fluorescence intensity at the cortex of epiblast cells was measured. The cell cortex was then segmented into two equal hemispheres and the fold of fluorescence accumulation in these two halves of the cell was measured. For each hemisphere, the integral of fluorescence intensity at the cortex was measured and compared to the integral of fluorescence intensity at the opposite hemisphere. Hemispheres were shifted along the cortex to find the maximum ratio (maximum asymmetry between two opposite halves).

To calculate the WT-Antxr2a and AAC-Antxr2a accumulation at the cap, the fluorescence intensity of WT-Antxr2a-eYFP or AAC-Antxr2a-eYFP at the plasma membrane of epiblast cells were measured and analyzed as previously described in ref. ^5^. In brief, the measured fluorescence intensity at the plasma membrane was fit to a Gaussian distribution (using MATLAB R2017b), using the following formula $f\left( x \right) = A{\mathrm{e}}\frac{{ - (x - \mu ^2)}}{{2\sigma ^2}} + O$ where *A* is the Amplitude, *µ* is the mean, *σ* is the standard deviation and *O* corresponds to the offset. These fits were used to calculate *A*, *µ*, *σ*, and *O*. The plane between *µ* and the center of the cell is the plane of the cap. This plane was used to determine the position of the cap with respect to the A/V axis of the embryo and to determine the angle between the position of the Antxr2a/F-actin caps and the mitotic plane. Therefore, the plane of the cap was calculated as the plane between the position of the maximal enrichment fluorescence intensity at the cortex of F-actin of Antxr2a and the cell center. The enrichment at the cap is calculated as (*A* + *O*)/*O*. To analyze significance between two independent samples, an unpaired *t*-test, two-tailed distribution was performed.

To determine the angle between the F-actin or Antxr2a cap and the embryonic axis, embryos were oriented with the animal pole up. Like this, the plane of the embryonic axis corresponds to the *y-*axis, while the plane of the cap is determined by *µ* in the Gaussian fit and the center of the cell. The angle between the F-actin or Antxr2a cap and the mitotic plane is the angle between the plane of the cap determined as before and the division plane at anaphase.

For all polar graphs shown in this work, we tested whether a given distribution is random, using the ratio of the observed angle to the expected (uniform) angle and *χ*^2^. If *χ*^2^ > *χ*^2^_critical_, the hypothesis is rejected and the probability that the distribution follows a random distribution is <5%.

### Fluorescence recovery after photobleaching (FRAP)

To address whether palmitoylation affects mobility of Antxr2a, either wild-type or *sptlc1* morphant embryos were injected with 75 pg of *wt-antxr2a-eYFP* mRNA in one cell out of eight to have mosaic expression. Wild-type embryos were also injected with 75 pg of *aac-antxr2a-eYFP* mRNA in one cell out of eight. Embryos developed until they reached the gastrula stage and mounted for imaging as described above. WT-Antxr2a-eYFP or AAC-Antxr2a-eYFP expressing epiblast cells were analyzed on an upright Zeiss 710 Multiphoton Confocal Microscope with a W-Plan-Apochromat 20×/1.0DIC M 27 70 mm lens. A region of 2.5 × 1 μm at the Antxr2a-eYFP cap was chosen and bleached using the full power of 488 nm laser. Recovery of the fluorescence was monitored for 300 s. Only cells whose fluorescence intensity profile fitted to a Gaussian distribution were considered for further analysis^5^. In those cells, fluorescence intensity of the bleached area was normalized by the fluorescence intensity of a non-bleached control area for each time point. Recovery kinetics were then fitted to an exponential function.

To address the half-life of Antxr2a, 1-cell stage embryos were injected with 100 pg of *antxr2a-eYFP* and dechorionated with 2 mg/ml Pronase (Roche applied Science) for 20 min. Dechorionated embryos were then transferred into 15 ml falcon tubes. Fish medium was removed and replaced by 1 ml Trypsin/EDTA. Embryos were agitated for 5 min at 37 °C. 100 μl of FBS was added. Embryos were centrifuged at 1000 × *g* for 3 min. Cells were resuspended in serum-free media and plated in Fibronectin-coated rings. The entire cell was bleached, and the fluorescence recovery was measured within 30 min, which is the time scale of the mitotic division in which oriented division is controlled. Fluorescence intensity of the bleached area was normalized by the fluorescence intensity of a non-bleached control area for each time point.

### Lipid extraction

For lipidomic analysis, 400 embryos were injected with either a control MO, 4 ng *wnt5a* MO or 0.5 ng *sptlc1* ATG MO. Morphant embryos were developed at 28 °C for 6 h. Embryos at shield stage were dechorionated and deyolked as described above, with the difference that for lipidomic analysis, the yolk was also kept and analyzed. Both deyolked embryos and yolk cells were stored at –80 °C. Pelleted cells were then resuspended in 100 μl H_2_O (LC-MS grade). Three-hundred sixty microliters of methanol (MS-grade) was added together with 10 μl internal lipid standards (DLPC (28:0 PC; 0.6 pM), DLPE (28:0 PE; 1.56 pM), DLPS (28:0 PS; 5.34 pM), 31:1 PI; 2.54 pM, C17 Ceramide (d18:1/17:0; 0.10 pM), C12 Sphingomyelin (d18:1/12:0; 5 pM), and C8 Glucosylceramide (d18:1/8:0; 0.20 pM)). Methyl-tert-butyl ether (MBTE) was used for total lipid extraction as described in ref. ^47^. Phase separation was induced by adding 200 μl H_2_O (LC-MS grade). The lower phase was washed again with 400 μl MBTE/Methanol/H_2_O (10:3:15). The two upper phases were then combined.

The total lipid extraction for the yolk cell was identical to the deyolked embryos, but without the additional resuspension in 100 μl H_2_O (LC-MS grade). For the total lipid extraction in 5 dpf *sptlc1*^*ug105*^ mutant embryos and siblings, embryos were first broken with 100 µl 1.4 mm zirconium oxide beads (Bertin Technologies) in 800 µl MS-grade H_2_O using a Precellys 24, lysis & homogenization machine (Bertin Technologies): three bursts of 45 s at 6200 r.p.m. with 45 s interruptions. Sphingolipid fraction for all the samples was obtained by mild alkaline hydrolysis of the total lipid fraction via treatment with methylamine followed by a butanol extraction.

### Targeted lipidomic analysis

Targeted analysis of lipid species was performed with Thermo Xcalibur 4.0 QF2 (ThermoFisher Scientific) using Multiple Reaction Monitoring (MRM) Mass Spectometry (MS)^20^. A Triversa NanoMate (Advion, Ithaca, NY, USA) was used to infuse samples onto a TSQ Vantage triple quadrupole mass spectrometer (ThermoFisher Scientific, Waltham, USA). MRM-MS was used to identify and quantify lipid species as previously described^48^. Data were converted and quantified relative to standard curves of internal standards that were incorporated to the samples before extraction. At least three independent biological replicates were analyzed, each of which was measured twice with three measurement cycles, leading to six technical replicates. Concentration values of lipid species were normalized to the total amount of inorganic phosphate as determined by colorimetric quantification with ammonium molybdate and ascorbic acid of pentachloroacetic acid hydrolyzed total lipid aliquots. To analyze the significance of the fold difference of each lipid species between two conditions, we performed an paired, two-tailed student’s *t*-test.

### Targeted analysis of sphingoid bases

To measure the levels of sphingoid bases (sphingosine and sphinganine) in *wnt5b* MO at 6 hpf and in *sptlc1*^*ug105/ug105*^ mutants at 4 dpf, 270 embryos injected with either a control MO or *wnt5b* MO and *sptlc1*^*ug105/ug105*^ mutants and their sibling were obtained. Morphant embryos were dechorionated and deyolked as described above. Both mutant and morphant embryos were then resuspended in 150 μl extraction buffer (ethanol:water:diethylether:pyridine(15:15:5:1) 2.1 10-3 M ammonium hydroxide), together with the internal standards (C17 sphingosine; 0.04 nmole; C17 sphinganine; 0.04 nmole). In all, 4 dpf sptlc1 mutant embryos were lysed on a Precellys 24, lysis & homogenization machine (Bertin Technologies) as described above. Extracts were cleared by centrifugation, dried under nitrogen, and frozen at –80 °C until further processing. Metabolites were resuspended in borate buffer (200 mM boric acid pH 8.8, 10 mM tris(2-carboxyethyl)phosphine, 10 mM ascorbic acid and 33.7 mM ^13^C_5_^15^N-valine), derivatized with 6-aminoquinolyl-hydroxysuccinimidyl carbamate (AQC, 2.85 mg/ml in acetonitrile) at 55 °C for 15 min and left at 24 °C overnight. Samples were analyzed using a reverse-phase C18 column (HPLC EC 100/2 Nucleoshell RP-18 2.7 mm) on an Accela high-performance liquid chromatography system (ThermoFisher Scientific, Waltham, MA), coupled to a TSQ Vantage triple quadrupole mass spectrometer (ThermoFisher Scientific, Waltham, USA) using multiple reaction monitoring.

### Isotope tracer-based metabolomics

To determine the activity of SPT, 40 one-cell stage embryos were injected with 0.5 nl of 1 M (^13^C_3_^15^N)-l-serine (heavy serine) and either control MO, *wnt5a* MO or *wnt5b* MO (as describe above). Embryos injected with 0.5 nl of 1 M (^13^C_3_^15^N)-l-serine (heavy serine) were also injected with 0.5 nl of 2 mM of the SPT inhibitor Myriocin (Sigma; reference # 476300) as a positive control. Embryos were developed at 28 °C until they reached shield stage (6hpf), dechorionated with 2 mg/ml Pronase (Roche applied Science) for 20 min and washed several times with fish medium. Embryos were then transferred to a 1.5 ml tube and deyolked as described above. The deyolked embryos were then processed to quantify isotopically labeled (^13^C_2_^15^N) sphingosine(^13^C_3_^15^N), following the above described targeted metabolomics protocol.

### Volcano plots

Lipid analyses were displayed using volcano plots, where the *y*-axis corresponds to the negative log10 of the *p*-value, while the *x-*axis corresponds to the log2 of the fold change between *wnt5b* morphants and control morphants, *wnt5a* morphant and control morphants, *sptlc1* morphants and control morphants, or *sptlc1*^*ug105/ug105*^ and siblings. In these volcano plots, points appearing to the left of the dash line correspond to lipid species that are downregulated either in *wnt5b*, *wnt5a*, *sptlc1a* morphants or in *sptlc1*^*ug105/ug105*^ mutants compared to the controls. On the contrary, dots appearing in the right side correspond to lipid species that are upregulated in the morphant/mutant conditions compared to the controls. The points found above the dashed, horizontal line are statistically significant.

### Treemap analysis

A Treemap is a space-filling 2-dimensional representation of hierarchical quantitative data^49^. Veronoi treemaps are an attractive alternative to the traditional rectangular treemaps, since they have a balanced and organic look. The veronoi treemaps used in this paper were computed using an in-house software, which implements an algorithm described by ref. ^50^. This algorithm produces veronoi diagrams, where the area of each veronoi cell approximately corresponds to the quantitative value of a lipid. We applied a natural hierarchical structure to the lipid data: lipid class, lipid subclass, number of double bonds, lipid identifier. The area of each parent cell is the sum of the areas of its children. The area of each lipid cell corresponds to the lipid quantity in nanomolar obtained by the mass spectrometry measurement. In order to allow an interactive comparison of the deyolked embryo and yolk lipidomes, we developed a web interface, which was implemented in java script based on the D3 (d3js.org) library.

### Data analysis and statistical analysis

GraphPad Prism version 8.0 and Excel version 16 softwares were used for statistical analyses of the data. Statistical analyses were performed using paired or unpaired student’s *t*-test. Differences were considered significant for *p*-value < 0.05. To test whether a given distribution is random, we used the chi-square test (see above and Supplementary Table 1). If *χ*^2^ > *χ*^2^_critical_, the hypothesis is rejected and the probability that the distribution follows a random distribution is <5%.

### Reporting summary

Further information on research design is available in the Nature Research Reporting Summary linked to this article.

## Supplementary information

Supplementary Information

Reporting Summary

Supplementary Movie 1

Supplementary Movie 2

Supplementary Movie 3

Supplementary Movie 4

Supplementary Movie 5

Supplementary Movie 6

Supplementary Movie 7

Supplementary Movie 8

Supplementary Movie 9

Supplementary Movie 10

Supplementary Movie 11

Supplementary Movie 12

Supplementary Movie 13

Supplementary Movie 14

Supplementary Movie 15

## Data Availability

The source data underlying all the figures of this study are available as Source Data files. The mass spectrometry lipidomic data have been deposited in a publicly available repository, with the following URLs: Supplementary Fig. 3 : [https://lipidomes.epfl.ch/exps/2201]; [https://lipidomes.epfl.ch/exps/2203]; Fig. 1: [https://lipidomes.epfl.ch/exps/2202]; [https://lipidomes.epfl.ch/exps/2204]; Fig. 3: [https://lipidomes.epfl.ch/exps/2207]; [https://lipidomes.epfl.ch/exps/2208]; Source data are provided with this paper.

## Source data

Source Data
