# Supplementary material for: Wnt-controlled sphingolipids modulate Anthrax Toxin Receptor palmitoylation to regulate oriented mitosis in zebrafish
Source: Nat Commun. 2020 Jul 3;11:3317. doi: 10.1038/s41467-020-17196-3 (PMC7335183; doi:10.1038/s41467-020-17196-3)
Supplement: Supplementary file 1 — Supplementary Information [file 41467_2020_17196_MOESM1_ESM.pdf]

SUPPLEMENTARY INFORMATION

Wnt-controlled sphingolipids modulate Anthrax Toxin Receptor palmitoylation to regulate oriented mitosis in zebrafish

Castanon, I. *§, Hannich, J.T. *, Abrami, L., Huber, F., Dubois, M., Müller, M., van der Goot, F.G., and Gonzalez-Gaitan, M. §

*. *These authors contributed equally to this work.*

§. *These authors jointly supervised this work*

Supplementary Fig. 1: Lipid profile of zebrafish embryos at gastrula stages.

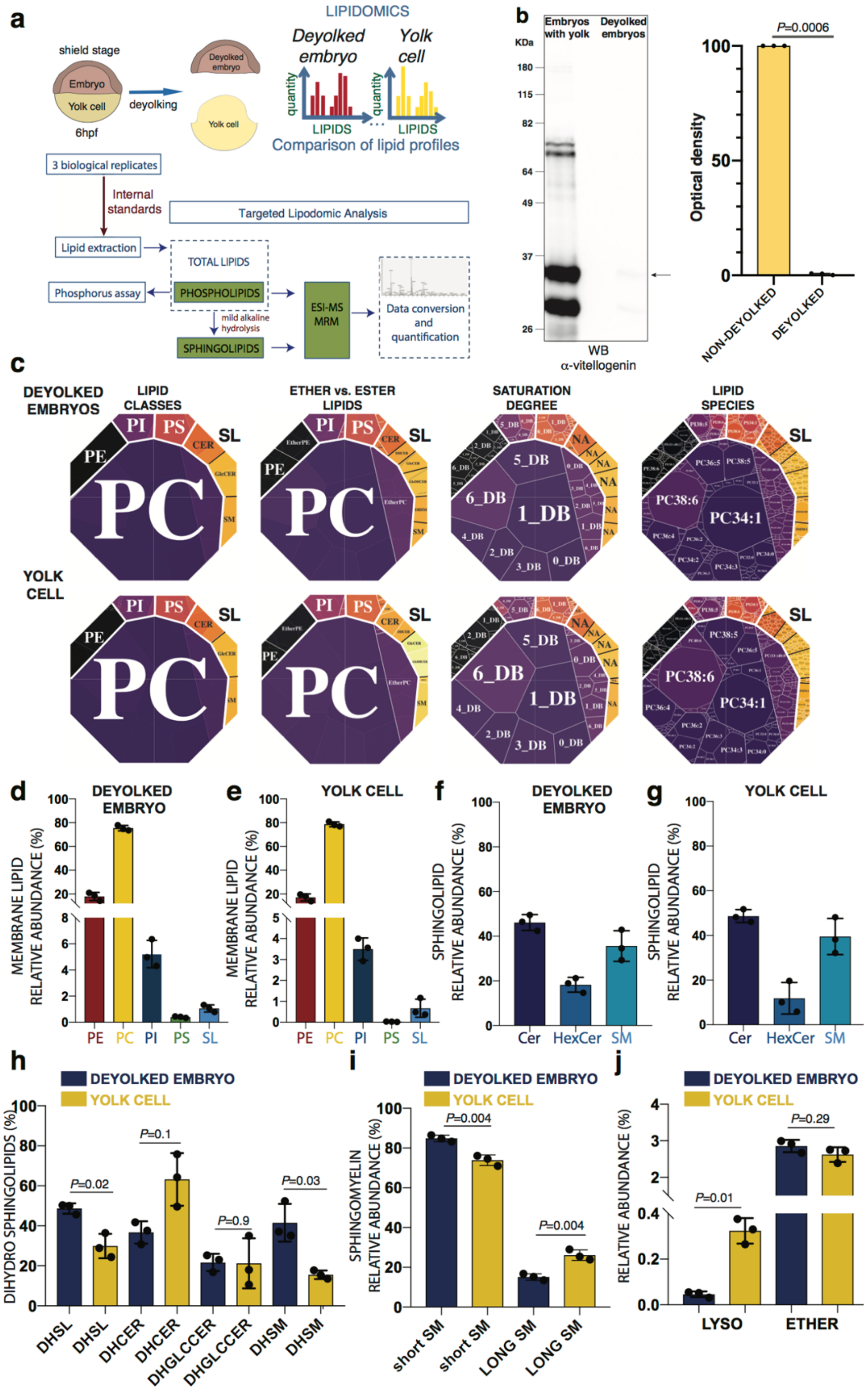

a Scheme of lipidomics workflow. Four hundred wild type embryos at gastrula stages were deyolked, and both the deyolked embryos and the yolk cells were kept for lipid analysis. Lipids from both deyolked embryos and yolk cells were extracted using Methyl-tert-butyl ether (MBTE). Three independent

biological samples per condition were prepared and mixed with internal standards. Total lipids were then extracted. From total lipid, one aliquot was used to perform phosphorus assay to normalize for extraction efficiency. A second aliquot was methylamine treated (mild alkaline hydrolysis) to remove phospholipids to be able to measure sphingolipids more efficiently. Phospholipids and sphingolipids were quantified *via* direct infusion ESI-MS MRM quantification. Data were converted and quantified based on standard curves. **b** Efficiency of yolk removal (deyolking) in 6hpf embryos. Embryos with yolk were analyzed by western blot against Vitellogenin and compared with deyolked embryos. Vitellogenin is the precursor of the major egg yolk protein in zebrafish[1]. It can therefore be used as a biomarker of yolk cell content[2]. Left, western blot against Vitellogenin showed that the yolk protein Vitellogenin was efficiently depleted in deyolked embryos. Right, quantification of the amount of Vitellogenin in deyolked embryos and embryos with yolk. Data are represented as the mean value +/- SEM from three independent experiments. Unpaired, 2-tailed student's t-test was used. **c** Interactive Treemap representations of the relative abundance of membrane lipids of deyolked embryos and yolk cells at gastrula stages (6hpf at 28°C), considering lipid classes (PC, PE, PI, PS and SL), ether *vs.* ester lipids, saturation degree and lipid species. Details can be visualized in Treemap [https://zebrafish-embryo-lipidome.vital-it.ch/#/]. Lipid amounts in **c** are normalized to the total amount of lipids. **d, e** Relative abundance of membrane lipid classes in both the deyolked embryo (**d**) and yolk cell (**e**). **f, g** Relative abundance of three main subclasses of sphingolipids: Cer, HexCer and SM in the deyolked embryo (**f**) and in the yolk cell (**g**). **h** Graph representing the relative abundance of dihydrosphingolipids (DHSL) normalized by the total amount of sphingolipids, as well as the relative amounts of dihydrosphingolipid subclasses normalized by the total amount of DHSL. **i** Relative abundance of short and long fatty acid chain sphingomyelin in deyolked embryo and yolk cell. Short chain: 10-18 fatty acid carbons; long chain: 20-26 fatty acid carbons. **j** Relative abundance of ether lipids and lysophospholipids normalized by the total amounts of lipids. Data in (**d**-**j**) are represented as the mean value +/- SD (standard deviation) from three independent experiments. Paired, 2-tailed student's t-test was used. Source data are provided as a Source Data file.

Supplementary Fig. 2: Epiblast cell divisions are randomized in *wnt5b* morphant embryos.

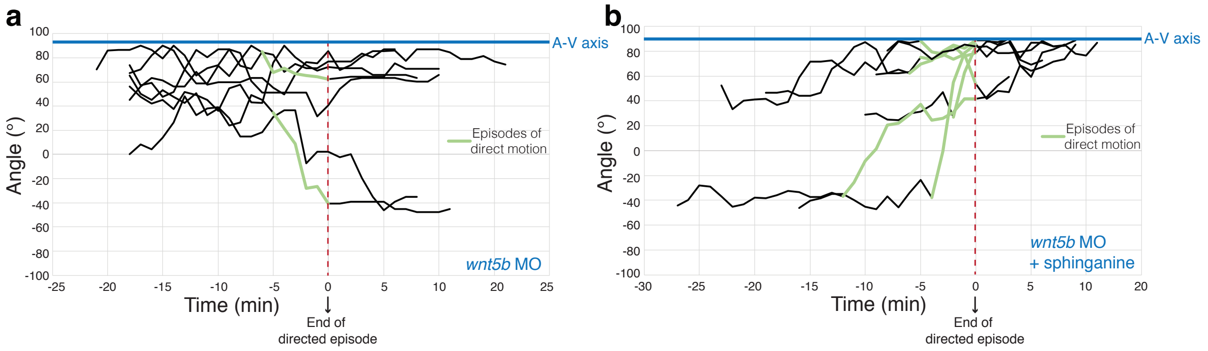

a, b Rotation of the mitotic spindle of epiblast cells visualized by the expression of doublecortin-GFP (dcx-GFP) in *wnt5b* morphant embryos (**a**; n=7 cells analyzed over 7 embryos; Supplementary Movie S12) and *wnt5b* morphants treated with sphinganine (**b**; n=7 analyzed over 7 embryos; Supplementary Movie S13). Episodes of the directed motion are shown in green. Blue line corresponds to the A-V axis of the embryo. We have previously shown that during division of wild type epiblast cells, the mitotic spindle is initially randomly oriented with respect to the A-V axis. Later, and coinciding with the formation of the F-actin and Antxr2a caps, the mitotic spindle is subjected to discrete episodes of directed rotation until it reaches the final position, which is aligned to the A-V axis[3]. Here, in *wnt5b* morphant embryos (**a**), the mitotic spindles fluctuate randomly (no episodes of directed motion could be found in most *wnt5b* morphant cells) and the trajectories of the spindle do not end aligned with the A-V axis. However, treatment of *wnt5b* morphants with sphinganine (**b**) recovered the orientation of the divisions: episodes of directed motion are now identified that end aligned with the A-V axis. Source data are provided as a Source Data file.

Supplementary Fig. 3: Epiblast cell divisions are randomized in *wnt5b* morphant embryos.

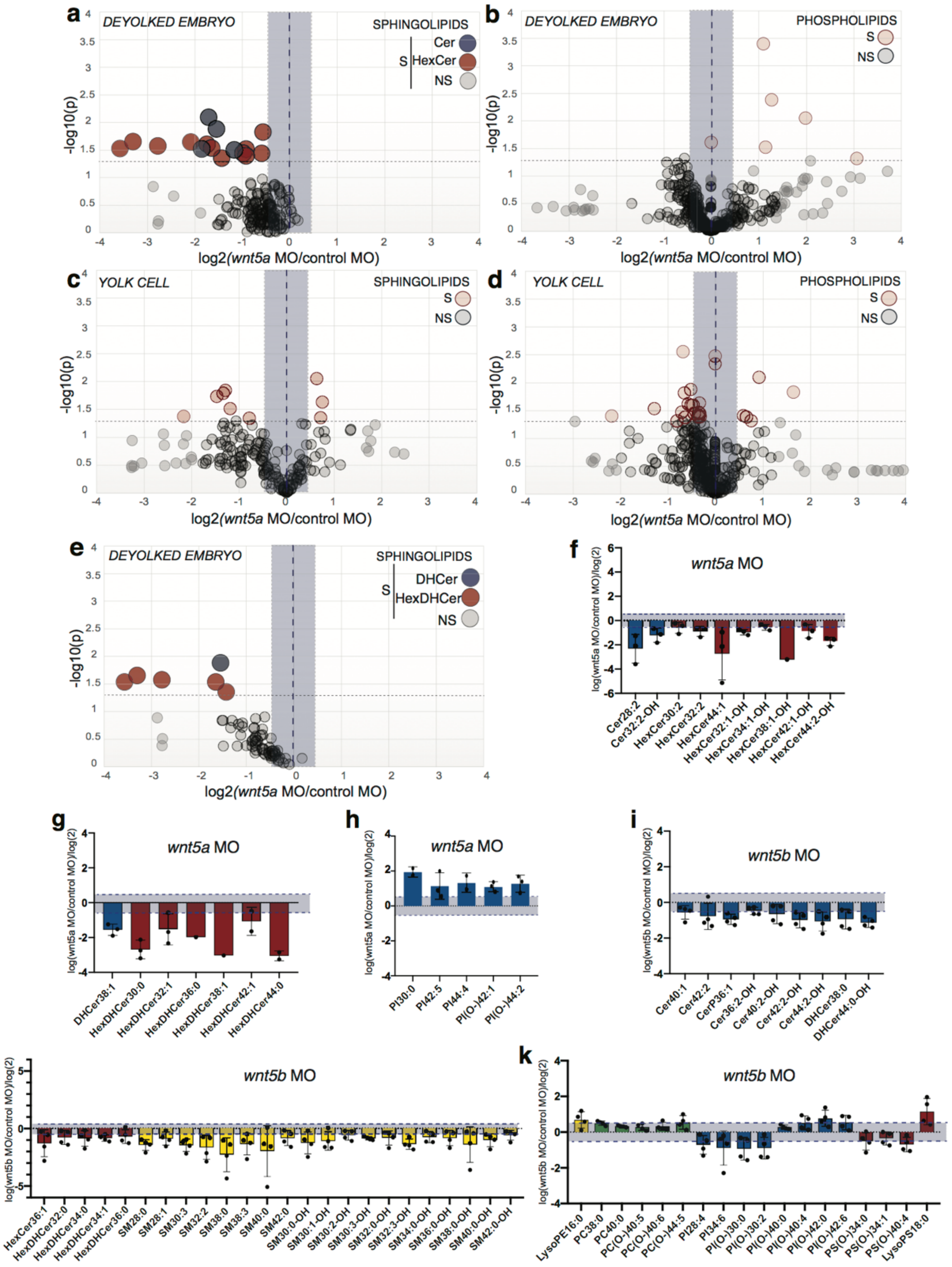

a-e Volcano plots of lipidomic data for *wnt5a* morphants compared to control embryos at gastrula stage (6hpf at 28°C) from three independent experiments. To determine whether differences for each lipid species between two conditions are statistically significant, an unpaired, 2-tailed student's t-test was used. Horizontal dashed lines threshold significance (*p*-value=0.05). NS (statistically Non Significant); S (statistically Significant). Blue shadowed area delimits +/-1.5-fold difference abundance with respect to control. Each dot corresponds to an individual lipid species. **a**, **c** Sphingolipids in deyolked embryos

(**a**) and yolk cell (**c**). Blue dots correspond to ceramides (Cer), red to Hexosylceramides (HexCer) and yellow to sphingomyelin (SM). **b**, **d** Phospholipids (PC, PE, PI, and PS) in deyolked embryos (**b**) and yolk cell (**d**). Note that the six phospholipids significantly upregulated beyond 1.5-fold in **b** correspond to phospholipids present at low levels (PI30:0 (1.0^{-4} Mol%), PI40:1 (4.0^{-4} Mol%), PI42:5 (2.6^{-3} Mol%), PI44:4 (3.5^{-4} Mol%), PI(O)42:1 (7.5^{-4} Mol%) and PI(O)44:2 (6.7^{-5} Mol%). **e** Volcano plot for sphingolipids in the *de novo* pathway (DHCer, dihydroceramide; HexDHCer, Dihydrohexosylceramides). Lipid amounts are normalized as pmol/nmol inorganic phosphate (see Methods). **f-k** Bar graphs representing either the fold difference of sphingolipids found statistically significant in deyolked *wnt5a* (Supplementary Fig. 2**a**, **e**) (**f**, **g**) and *wnt5b* morphants (Fig. 1**a**) (**i**, **j**), or the fold difference of phospholipids found statistically significant in deyolked *wnt5a* (Supplementary Fig. 2**b**) (**h**) and *wnt5b* morphants (Fig. 1**b**) (**k**). Blue shadowed area delimits +/-1.5-fold difference abundance with respect to control. Error bars correspond to the standard deviation of the mean of the fold difference for each lipid species represented. Note that the amounts of sphingolipids that are significantly downregulated in *wnt5b* morphants are 10 times larger (0.15% of total lipids) than in the case of *wnt5a* morphant embryos (0.018% of total lipids). Source data are provided as a Source Data file.

Supplementary Fig. 4: *sptlc1* pattern of expression and mutant phenotype.

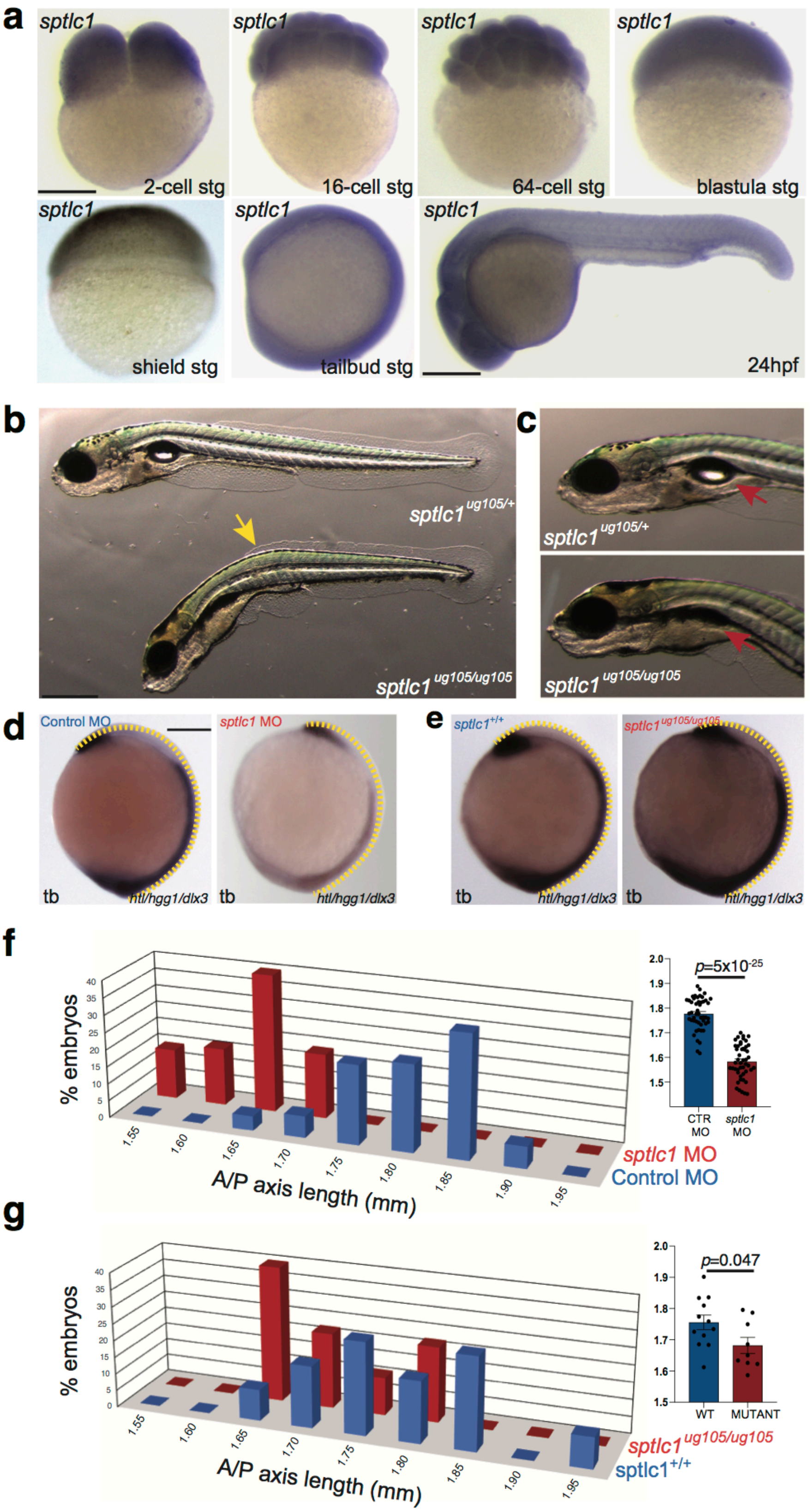

a Pattern of expression of *sptlc1* during early zebrafish development. Whole mount *in situ* hybridization of *sptlc1* antisense riboprobe was performed twice at different stages of embryonic development. *sptlc1*

transcripts can be ubiquitously detected during the first 24 hours of development. **b, c** Representative images of 4dpf *sptlc1*$^{ug105/+}$ and *sptlc1*$^{ug105/ug105}$ embryos. Homozygous embryos showed a decrease in size, curved trunk (yellow arrow in **b**), and failed to inflate the swim bladder (red arrow in **c**). These phenotypes were reproducibly observed in more than 10 independent experiments. **d**, **e** Lateral view of representative tail bud stage *sptlc1* morphant (**d**) or *sptlc1*$^{ug105/ug105}$ mutant (**e**) embryos analyzed by *in situ* hybridization for the expression of *no tail* (*ntl*) in the notochord, *hatching gland 1* (*hgg1*) in the prechordal plate and *dlx3* in the neural plate. Dashed yellow lines correspond to the length of the body axis (A/P axis). **f**, **g** Histogram of the anterior-posterior (A/P) axis length from (**d**, **e**) showing the extension along the body axis in embryos injected with either *sptlc1* morpholino (n=48 embryos; red) or control morpholino (n=48 embryos; blue) (**f**) or in *sptlc1*$^{ug105/ug105}$ mutant embryos (n=9 embryos; red) compared to their wild type siblings (n=12 embryos; blue) (**g**). Note that in conditions defective for *sptlc1,* the notochord length is shorter. Unpaired, 2-tailed student's t-test comparing the average between *sptlc1* and control morphants or between wild type and *sptlc1* mutants (bar graphs on the right) was used. In these graphs, error bars represent the mean value +/- SEM. Scale bars: 250µm. Source data are provided as a Source Data file.

Supplementary Fig. 5: Sphingolipids are downregulated in *sptlc1* impaired embryos.

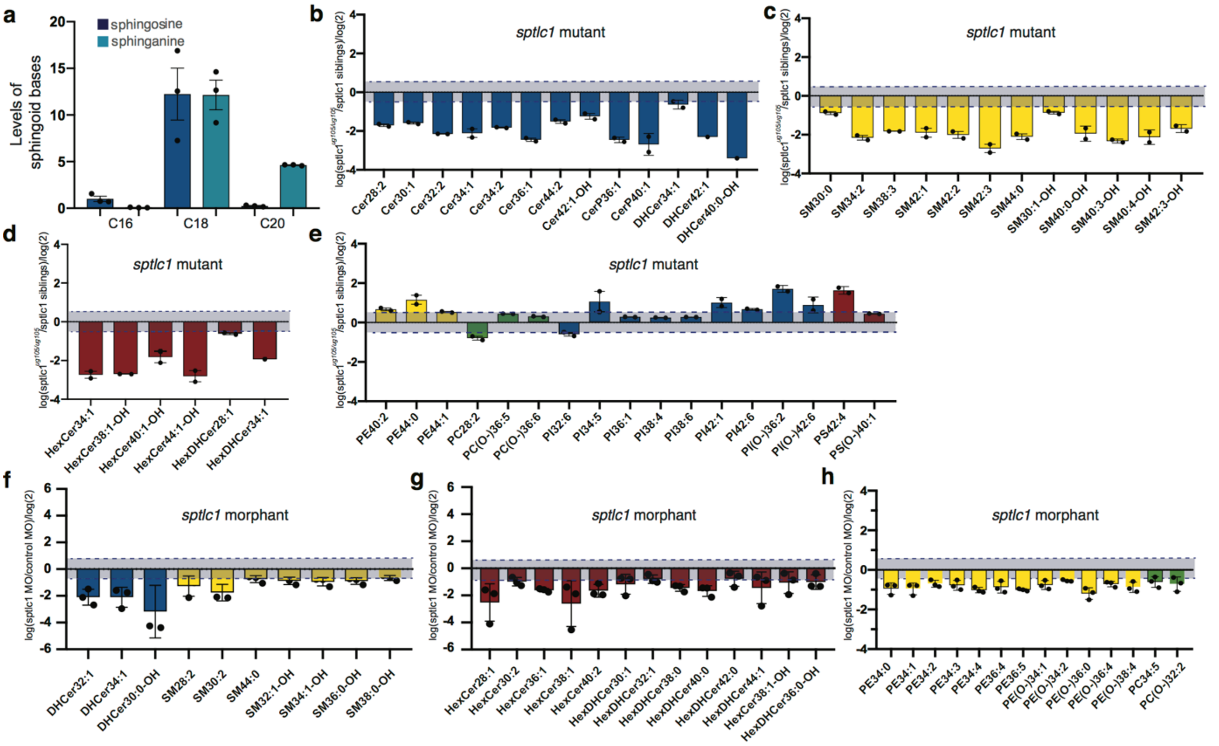

a Levels of the shingoid bases (sphingosine and sphinganine) containing 16, 18 and 20 carbons (C16, C18, and C20) in wild type embryos at gastrula stages from three independent biological samples. The most abundant alkyl chain length for both sphingoid bases is 18. Therefore, for further analysis of sphingoid bases, we focus on the ones with akyl chain length 18. Data are represented as mean value +/- SEM. **b-d, f**, **g** Bar graphs representing the fold difference of sphingolipids found statistically significant in *sptlc1*$^{ug105/ug105}$ mutant embryos (Fig. 2**e**) (**b**-**d**) and in deyolked *sptlc1* morphants (Fig. 3**c**) (**f**, **g**). All the SL subclasses and SL formed *via* the *de novo* pathway (dihydrosphingolipids) are downregulated in both *sptlc1*$^{ug105/ug105}$ mutants (**b-d**) and *sptlc1* morphants (**f**, **g**). **e**, **h** Bar graphs representing the fold difference of phospholipids found statistically significantly altered in *sptlc1*$^{ug105/ug105}$ mutant embryos (Fig. 3**f**) (**e**) or in deyolked *sptlc1* morphants (Fig. 3**d**) (**h**). Note that the fold difference of most phospholipids found statistically significantly altered in *sptlc1*$^{ug105/ug105}$ mutant embryos is below 1.5, with the exception of minor PI species, whose amount cannot therefore be precisely determined. Note that the reduction in the levels of PE in deyolked *sptlc1* morphant embryos is probably a secondary effect of the decrease in the levels of sphingolipids. As the levels of SL are decreased, the amount of SL available that are degraded to form PE are also reduced, resulting in a decrease in the total levels of PE[4]. Blue shadowed area delimits +/-1.5-fold difference abundance with respect to control. Error bars correspond to the standard deviation of the mean of the fold difference for each lipid species represented. Lipid amounts are normalized as pmol/nmol inorganic phosphate (see Methods). Source data are provided as a Source Data file.

Supplementary Fig. 6: Sphinganine restores oriented divisions in *sptlc1* morphants.

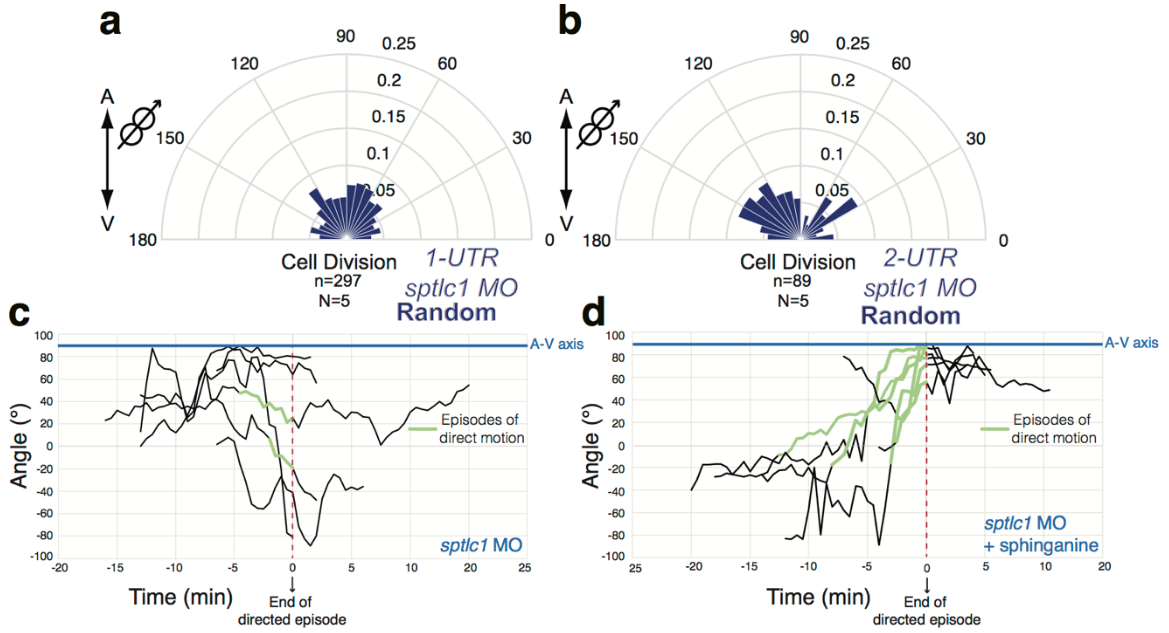

a, **b** Polar graphs showing the orientation of division of epiblast cells with respect to the A/V embryonic axis (represented by a bi-directional arrow on the right) in embryos injected with two independent *sptlc1* UTR morpholinos. Injections of embryos with either 2ng of *sptlc1* UTR MO-1 (**a**), or 2ng of *sptlc1* UTR MO-2 (**b**) results in randomization of divisions of epiblast cells. n (number of cell divisions analyzed) over N (number of embryos). χ^2 test was used (see Supplementary Table 1). **c**, **d** Rotation of the mitotic spindle of epiblast cells visualized by the expression of doublecortin-GFP (dcx-GFP) in *sptlc1* morphant embryos (**c**; n=6 cells analyzed over 6 embryos; Supplementary Movie S14) and *sptlc1* morphants treated with sphinganine (**d**; n=7 cells analyzed over 7 embryos; Supplementary Movie S15). Episodes of the directed motion are shown in green. Blue line corresponds to the A-V axis of the embryo. No episodes of directed motion could be found in most *sptlc1* morphant epiblast cells. Therefore, the rotation of the mitotic spindle fluctuates randomly and does not end aligned with the A-V axis. Treatment of *sptlc1* morphants with sphinganine (**d**) results in trajectories of the mitotic spindle showing episodes of directed motion that end aligned with the A-V axis. Source data are provided as a Source Data file.

Supplementary Fig. 7: Palmitoylation sites are conserved in zebrafish Antxr2a.

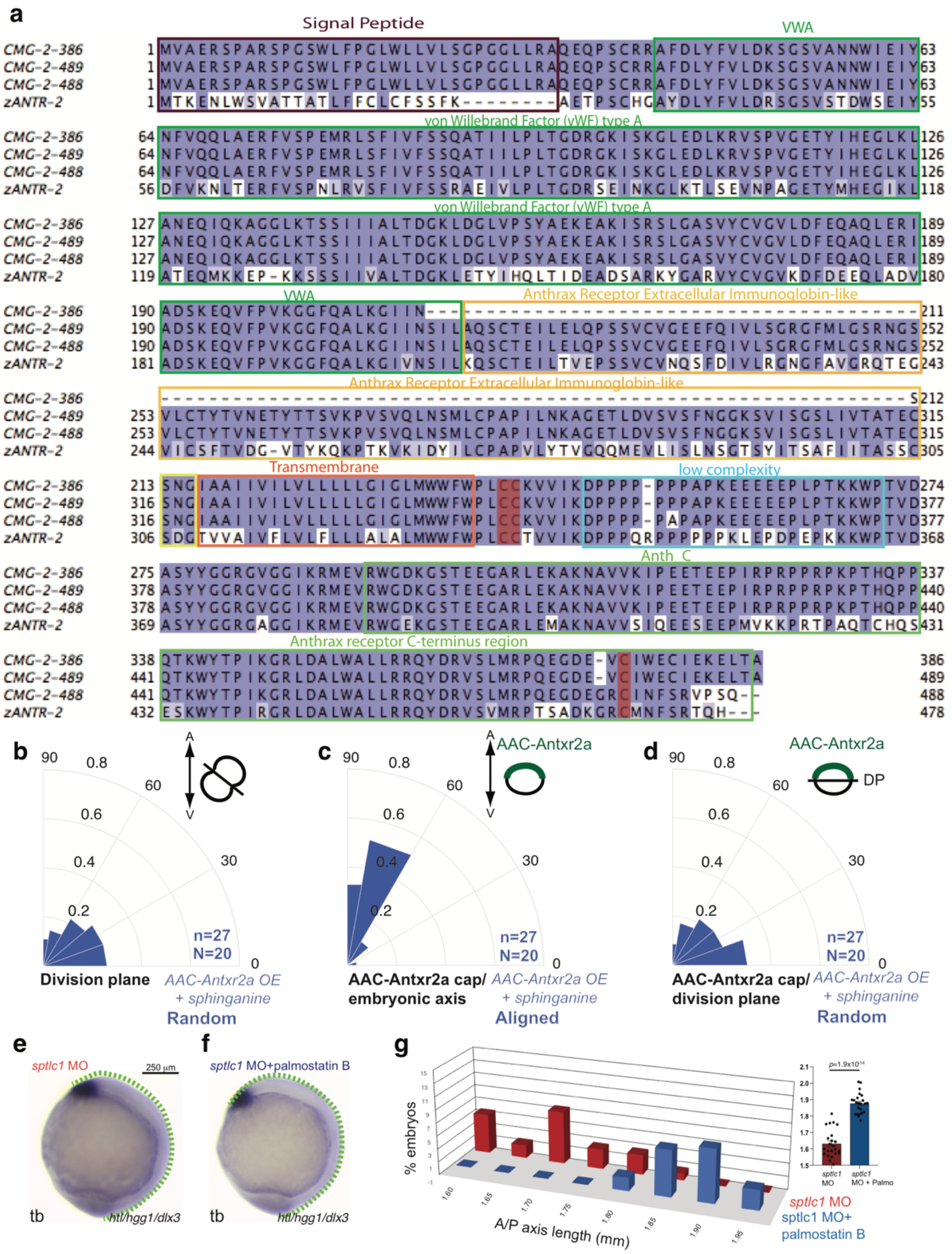

a Sequence alignment (MUSCLE algorithm with default settings[5]) of the zebrafish Anthrax Toxin Receptor 2a and the human TEM8/ANTXR1 and CMG2/ANTXR2. Zebrafish Antxr2a contains three conserved cysteine residues in its cytoplasmic tail, which are potential sites for S-palmitoylation (Cys-334, Cys-335 and Cys-470; highlighted in pink)[6]. **b-d** Polar graphs showing the frequency distribution of the division plane (**b**), the angle between the division axis and the A/V embryonic axis (**c**) and the

angle between the AAC-Antxr2a and the division plane in AAC-Antxr2a expressing embryos and treated with sphinganine. Randomization of division in AAC-Antxr2a expressing epiblast cells cannot be rescued by addition of sphinganine. χ^2 test was used (see Supplementary Table 1). n (number of cells analyzed) over N (number of embryos). **e**, **f** Lateral view of representative tail bud stage *sptlc1* morphants (**d**) or *sptlc1* morphants treated with palmostatin B (**e**) analyzed by *in situ* hybridization for the expression of *no tail* (*ntl*) in the notochord, *hatching gland 1* (*hgg1*) in the prechordal plate and *dlx3* in the neural plate. Dashed green lines correspond to the length of the body axis (A/P axis). **g** Histogram of the A/P axis length from (**e**, **f**) showing the extension along the body axis in embryos injected with *sptlc1* morpholino alone (n=24 embryos; red) or additionally treated with palmostatin B (n=23 embryos; blue). Note that the length of the AP axis is restored in *sptlc1* morphants treated with palmostatin B. For statistical analysis, an unpaired, 2-tailed student's t-test comparing the average between the two conditions (bar graphs on the right) was used. In this graph, error bars represent the mean value +/- SEM. Source data are provided as a Source Data file.

Supplementary Fig. 8: Mobility, stability and protein interaction is not affected in AAC-Antxr2a.

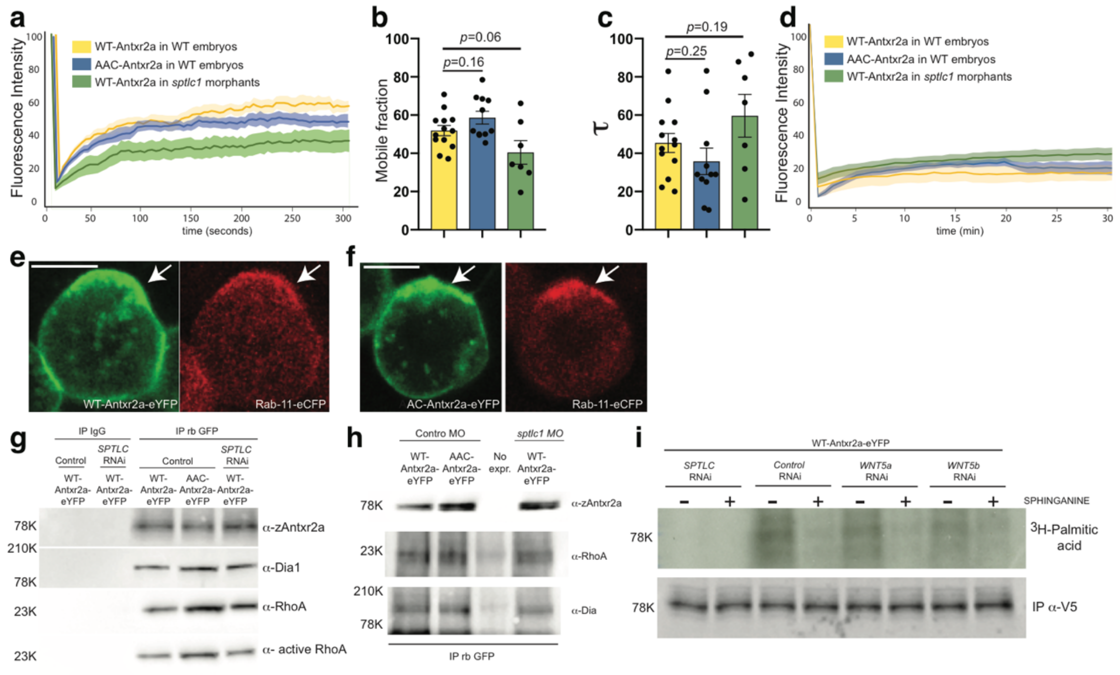

a-d Fluorescence recovery after photobleaching (FRAP) experiments. Control morphants were injected with either WT-Antxr2a-eYFP (n=13 cells) or AAC-Antxr2a-eYFP (n= 11 cells). *sptlc1* morphants were injected with WT-Antxr2a-eYFP (n= 7 cells). (**a**) Curve of fluorescence recovery after bleaching a region of the WT-Antxr2a-eYFP or AAC-Antxr2a-eYFP cap. **b** Graph representing the mobile fraction calculated from FRAP in (**a**). **c** Graph representing the time at which half of the fluorescence has recovered (τ) from FRAP in (**a**). Data are represented as the mean value +/- SEM. Unpaired, 2-tailed student's t-test was used. Neither the mobile fraction nor τ change in the palmitoylated-defective Antxr2a (AAC-Antxr2a) or in *sptlc1* morphant embryos compared to the control WT-Antxr2a. **d** Curve of fluorescence recovery after bleaching the entire epiblast cell of control morphants injected with either WT-Antxr2a-eYFP (n=6 cells) or AAC-Antxr2a-eYFP (n= 5 cells), or in *sptlc1* morphants injected with WT-Antxr2a-eYFP (n= 5 cells) to calculate the half-life of the receptor. There is very little fluorescence recovery within 30 minutes, indicating that the half-life of the receptor is much longer than the time scale for the mitotic division of epiblast cells (around 30 minutes). **e**-**f** Representative confocal images from 5 independent experiments showing the colocalization between Rab11 and WT-Antxr2a (**e**) and Rab 11 and AAC-Antxr2a (**f**). Scale bars: 10 μm. **g** Representative blot from immunoprecipitation of Antxr2a and RhoA and Dia expressed in HeLa cells from three independent experiments. Control HeLa cells were transfected with either wild type Antxr2a-eYFP or with the palmitoylation defective mutant form of Antxr2a (AAC-Antxr2a-eYFP), whereas HeLa cells treated with SPTLC RNAi were transfected with WT-Antxr2a-eYFP. Immunoprecipitation was performed using an anti-GFP antibody or unspecific IgG as a control. RhoA, Dia and co-immunoprecipitated Antxr2a were detected using anti-RhoA, anti-GPD-bound RhoA, anti-Dia and anti-Antxr2a antibodies, respectively. **h** Representative blot from

immunoprecipitation of Antxr2a and RhoA and Dia in zebrafish embryos from three independent experiments. Zebrafish embryos were injected with 0.1 μg/μl of either WT-Antxr2a-eYFP or AAC-Antxr2a-eYFP in control morpholino injected embryos or with WT-Antxr2a-eYFP in *sptlc1* morpholino injected embryos. As before, immunoprecipitation was performed using an anti-GFP antibody and detection was performed using anti-RhoA, anti-Dia and anti-Antxr2a antibodies. (**i**) Representative plot from three independent experiments showing the levels of ^{3}H-palmitate incorporation in WT-zAntxr2a expressed in either SPT RNAi, WNT5a RNAi or WNT5b RNAi HeLa cells with or without sphinganine. Note that feeding HeLa cells with both sphinganine prevents incorporation of ^{3}H-palmitate. Both sphinganine and ^{3}H-palmitate use the same uptake machinery[7]. Therefore, we cannot monitor palmitoylation of the receptor when feeding sphinganine to the cells. Source data are provided as a Source Data file.

Supplementary Table 1: Chi-square test to address randomization of cell division

X^2 (degrees of freedom, *n* = sample size) = chi-square statistic value, *p* = *p* value

**Figure 2h**	X^2 (17, *n* = 334) = 25.08, *p* = .05	Randomized
**Figure 2i**	X^2 (17, *n* = 204) = 43.52, *p* = .05	Aligned
**Figure 2j**	X^2 (17, *n* = 342) = 24.32, *p* = .05	Randomized
**Figure 2k**	X^2 (17, *n* = 350) = 126.95, *p* = .05	Aligned
**Figure 4b**	X^2 (17, *n* = 275) = 161, *p* = .05	Aligned
**Figure 4c**	X^2 (17, *n* = 275) = 231.55, *p* = .05	Aligned
**Figure 4d**	X^2 (17, *n* = 298) = 22.98, *p* = .05	Randomized
**Figure 4e**	X^2 (17, *n* = 504) = 382.57, *p* = .05	Aligned
**Figure 4f**	X^2 (17, *n* = 225) = 18.76, *p* = .05	Randomized
**Figure 4g**	X^2 (17, *n* = 409) = 144.16, *p* = .05	Aligned
**Figure 5d left**	X^2 (8, *n* = 24) = 79.5, *p* = .05	Aligned
**Figure 5d right**	X^2 (8, *n* = 22) = 50.82, *p* = .05	Aligned
**Figure 5g left**	X^2 (8, *n* = 27) = 80, *p* = .05	Aligned
**Figure 5g right**	X^2 (8, *n* = 20) = 40.3, *p* = .05	Aligned
**Figure 6a left**	X^2 (8, *n* = 24) = 84, *p* = .05	Aligned
**Figure 6a right**	X^2 (8, *n* = 22) = 7.45, *p* = .05	Randomized
**Figure 6b left**	X^2 (8, *n* = 24) = 132, *p* = .05	Aligned
**Figure 6b right**	X^2 (8, *n* = 22) = 9.09, *p* = .05	Randomized
**Figure 6c left**	X^2 (8, *n* = 27) = 102.67, *p* = .05	Aligned
**Figure 6c right**	X^2 (8, *n* = 20) = 4.30, *p* = .05	Randomized
**Figure 6d left**	X^2 (8, *n* = 27) = 124, *p* = .05	Aligned
**Figure 6d right**	X^2 (8, *n* = 20) = 3.4, *p* = .05	Randomized
**Figure 7g**	X^2 (17, *n* = 400) = 14.70, *p* = .05	Randomized
**Figure 7h**	X^2 (17, *n* = 602) = 113.13, *p* = .05	Aligned
**Figure 7j**	X^2 (8, *n* = 23) = 7.13, *p* = .05	Randomized
**Figure 7k**	X^2 (8, *n* = 23) = 58, *p* = .05	Aligned

**Figure 7l**	X^2 (8, n = 23) = 7.91, p = .05	Randomized
**Figure 7m**	X^2 (8, n = 28) = 22.78, p = .05	Randomized
**Supplementary Figure 6a**	X^2 (17, n = 297) = 26.5, p = .05	Randomized
**Supplementary Figure 6b**	X^2 (17, n = 89) = 17.99, p = .05	Randomized
**Supplementary Figure 7b**	X^2 (8, n = 27) = 2.67, p = .05	Randomized
**Supplementary Figure 7c**	X^2 (8, n = 27) = 36.67, p = .05	Aligned
**Supplementary Figure 7d**	X^2 (8, n = 27) = 4, p = .05	Randomized

Supplementary Table 2: List of primers, siRNAs and morpholinos

**To genotype sptlc1 ug105 mutants**	
**G-Sptlc1-Forward**	5'-GCAGCCTATATTAAATGTTC-3'
**G-Sptlc1-Revese**	5'-ACAGCCGAATACAACCACCT-3'
**To generate pCS2-AAC-Antxr2a-eYFP**	
**AAC-Forward**	5'-GGTCTTTAATAACGACAGTGGCGGCTAGAGGCCAGAACCACCACATCA-3'
**AAC-Reverse**	5'-TGATGTGGTGGTTCTGGCCTCTAGCCGCCACTGTCGTTATTAAAGACC-3'
**To clone zebrafish Sptlc1 cDNA**	
**Sptlc1-Forward**	5'-CGGAATTCATGGCGTCGGGGCAACAGTG-3'
**Sptlc1-Reverse**	5'-CCGCTCGAGTCATTTCAGGACGTGTAAAG-3'
**To generate zebrafish Sptlc1 RNA probe**	
**I-Sptlc1-Forward**	5'-ATTGAAGATCAGAAGAATCCGCGTA-3'
**I-Sptlc1-Reverse**	5'- **AATTTAATACGACTCACTATAGG**CGAGCCAACGTTAGTGCGACCTGTC-3'
**siRNAs**	
**glycoprotein VSV-G**	5'-ATTGAACAAACGAAACAAGGA-3
**WNT5a**	5'-CCGGATAACCTTGTAACATAT-3'
**WNT5b**	5'-GTTTATCATCGGTGCCCAGCC-3'
**SPTLC1**	5'-CAAGAACGATCTGATCTTACA-3'
**SPTLC2**	5'-AAGGAGGTTGTGGTAACTAAA-3'
**Morpholinos**	
***sptlc1*-1-UTR MO**	5'-GCGACTTCCTGATTGATAAAGAGGC-3'

***sptlc1*-2-UTR MO**	5'-CGCCATTTTGACTGAGTCACATGAT-3'
***sptlc1*-ATG MO**	5'-ACCCACTGTTGCCCCGACGCCATTT-3'
***wnt5a* MO**	5'-TCCACTTCAGCTTCAGCAGCATCAT-3'
***wnt5b* MO**	5'-GTCCTTGGTTCATTCTCACATCCAT-3'

Supplementary References

1. Zhong, L. *et al.* Distribution of vitellogenin in zebrafish (Danio rerio) tissues for biomarker analysis. *Aquat Toxicol* **149**, 1-7 (2014).
2. Link, V., Shevchenko, A. & Heisenberg, C.P. Proteomics of early zebrafish embryos. *BMC Dev Biol* **6**, 1 (2006).
3. Castanon, I. *et al.* Anthrax toxin receptor 2a controls mitotic spindle positioning. *Nat Cell Biol* **15**, 28-39 (2013).
4. Rodriguez-Cuenca, S., Pellegrinelli, V., Campbell, M., Oresic, M. & Vidal-Puig, A. Sphingolipids and glycerophospholipids - The "ying and yang" of lipotoxicity in metabolic diseases. *Prog Lipid Res* **66**, 14-29 (2017).
5. Edgar, R.C. MUSCLE: multiple sequence alignment with high accuracy and high throughput. *Nucleic Acids Res* **32**, 1792-1797 (2004).
6. Abrami, L., Leppla, S.H. & van der Goot, F.G. Receptor palmitoylation and ubiquitination regulate anthrax toxin endocytosis. *J Cell Biol* **172**, 309-320 (2006).
7. Narita, T., Naganuma, T., Sase, Y. & Kihara, A. Long-chain bases of sphingolipids are transported into cells via the acyl-CoA synthetases. *Sci Rep* **6**, 25469 (2016).
